# Volatile Natural Deep Eutectic Solvents (VNADESs) for Extraction of Shikonin Derivatives from *Echium vulgare* Roots and Evaluation of Biological Activity

**DOI:** 10.3390/molecules31091434

**Published:** 2026-04-26

**Authors:** Magdalena Kulinowska, Sławomir Dresler, Agnieszka Grzegorczyk, Martyna Zagórska-Dziok, Aleksandra Ziemlewska, Wirginia Kukula-Koch, Katarzyna Sawa-Wejksza, Maciej Strzemski

**Affiliations:** 1 Doctoral School, Medical University of Lublin, 7 Chodzki St., 20-093 Lublin, Poland; 2 Department of Analytical Chemistry, Medical University of Lublin, 4A Chodzki Str., 20-093 Lublin, Poland; slawomir.dresler@umlub.edu.pl; 3 Department of Plant Physiology and Biophysics, Institute of Biological Sciences, Faculty of Biology and Biotechnology, Maria Curie-Skłodowska University, 19 Akademicka St., 20-033 Lublin, Poland; 4 Department of Pharmaceutical Microbiology, Medical University of Lublin, 1 Chodzki Str., 20-093 Lublin, Poland; agnieszka.grzegorczyk@umlub.edu.pl; 5 Department of Technology of Cosmetic and Pharmaceutical Products, Medical College, University of Information Technology and Management in Rzeszow, 2 Sucharskiego St., 35-225 Rzeszow, Poland; mzagorska@wsiz.edu.pl (M.Z.-D.); aziemlewska@wsiz.edu.pl (A.Z.); 6 Department of Pharmacognosy with Medicinal Plants Garden, Medical University of Lublin, 20-093 Lublin, Poland; virginia.kukula@gmail.com; 7 Department of Virology and Immunology, Institute of Microbiology and Biotechnology, Maria Curie-Skłodowska University, 19 Akademicka St., 20-033 Lublin, Poland; katarzyna.sawa-wejksza@mail.umcs.pl

**Keywords:** green chemistry, nitric oxide, tyrosine kinase inhibitors, antimicrobial activity, anti-inflammatory activity, methicillin-resistant *Staphylococcus aureus*, *Candida auris*, *Klebsiella pneumoniae*, *Pseudomonas aeruginosa*, *Acinetobacter baumannii*

## Abstract

**Background:** Shikonins are natural naphthoquinones that exhibit a range of biological activities. They are typically extracted using nonpolar solvents; however, green extraction approaches remain underexplored. **Methods:** Phytochemical profiling of *E. vulgare* root extracts was performed using HPLC-ESI-QTOF-MS/MS and quantitative analysis using HPLC-PDA. Shikonin extraction was performed using VNADESs based on thymol, camphor, menthol and benzyl alcohol. The feasibility of removing the VNADES from the extracts via freeze-drying was assessed. The cytotoxic, antioxidant, anti-inflammatory and antimicrobial activities of the hexane extract and the selected VNADES-based extract (TBa 2:8) were compared. **Results:** Eight shikonin derivatives were identified in the extracts. VNADES extracts contained comparable amounts of shikonin to hexane extracts; however, freeze-drying resulted in significant shikonin content loss. TBa 2:8 extract exhibited noticeably lower cytotoxicity than the hexane extract while its antioxidant potential depended on the assay applied. In contrast to the hexane extract, TBa 2:8 demonstrated the ability to reduce intracellular ROS and NO levels. However, the hexane extract exhibited stronger antimicrobial activity. **Conclusions:** VNADES systems enable efficient extraction of shikonin derivatives with performance comparable to hexane. Although the resulting extracts exhibit multidirectional biological activity, it remains challenging to remove the VNADESs effectively without losing the shikonins.

## 1. Introduction

Shikonin (hydroxy-1,4-naphthoquinone) is a secondary metabolite found in species belonging to the genera *Alkanna*, *Lithospermum*, *Echium, Onosma, Arnebia, Buglossoides, Craniospermum, Cynoglossum, Nonea, Stenosolenium*, *Eritricium,* and *Anchusa,* all of which are members of the Boraginaceae family [1,2,3]. Shikonin and its derivatives are characterized by their bright red-purple pigmentation [1]. The presence of the red pigment in the external layer of the *Alkanna tinctoria* roots likely contributed to early botanical interest in this compound [3]. Shikonin has been used for millennia in traditional Chinese medicine to treat conditions such as burns, carbuncles, measles, macular eruptions, and sore throat [4].

In 1976, Papageorgiou validated earlier reports regarding the wound-healing and antimicrobial properties of *Alkanna tinctoria* root extracts and was the first to identify alkannin derivatives [3]. These findings prompted further researches into the phytochemical composition of this species. Shikonin and alkannin are enantiomeric naphthoquinones, with various derivatives primarily formed by esterification of the side-chain hydroxyl group. These include acetylshikonin, isobutylshikonin, isovalerylshikonin, *α*-methylbutyrylshikonin, *β,β*-dimethylacrylshikonin, teracrylshikonin, angelylshikonin, *β*-hydroxyisovalerylshikonin, *β*-acetoxyisovalerylshikonin, propionylshikonin, 1-methoxyacetylshikonin, benzoylshikonin, 2-methyl-hexa-1′,3′-dienoyl shikonin, and butylshikonin. Each shikonin ester forms a chiral pair with its corresponding alkannin ester. Other natural derivatives of shikonin/alkannin include arnebin-2 and its ester: acetylarnebin-2, arnebin-5, arnebin-6, and lithospermidin-B [5,6].

Numerous pharmacological properties of shikonin have been elucidated through various signaling pathways and mechanisms of action. Shikonin demonstrates antioxidant activity, providing neuroprotective and anti-inflammatory effects, particularly in cardiovascular diseases and rheumatoid arthritis [3]. Additionally, it exhibits anti-ischemic, anti-cancer, anti-tumor, antiviral, anti-acne, and anti-ulcer properties It is also used in the treatment of burns, eczema, and wound healing, as well as for therapeutic interventions in conditions such as endometriosis, fever reduction, detoxification, and ailments including measles, carbuncles, hepatitis, vaginitis, cervicitis, empyrosis, pathopyretic ulcers, and purpura eczema [1,7]. Researchers highlight the promising potential of shikonin and its derivatives as effective therapeutic agents in oncology, with particular focus on their application in the treatment of non-small-cell lung and colorectal carcinomas [4,8], especially since some shikonins are very potent inhibitors of tyrosine kinases [9]. Shikonins exhibit strong antimicrobial properties [10], which is important given the growing resistance of microorganisms to antibiotics. Finding effective agents to combat infections caused by methicillin-resistant *Staphylococcus aureus* [11], *Candida auris* [12], *Klebsiella pneumoniae* [13], *Pseudomonas aeruginosa* [14] and *Acinetobacter baumannii* [15] is a major challenge for modern science.

Although the medical properties of shikonin are well established and its extraction methodologies have been thoroughly documented [1], the selection of solvents remains a challenge, particularly regarding safety for laboratory workers. In addition to safety concerns, conventional solvents may also contribute to the degradation of sensitive naphthoquinones. The stability of shikonin derivatives has been shown to depend on environmental conditions, including the nature of the solvent, which may influence the rate of photochemical degradation [16].

Shikonin is characterized by its limited solubility in water [17], which necessitates the utilization of hydrophobic solvents for efficient extraction. Commonly employed solvents include methanol, ethanol, and, most frequently, n-hexane [1,7,18]. Historically, n-hexane was widely used as a universal solvent until its toxicity to the peripheral nervous system, specifically as a delayed distal polyneuropathy, was identified [19]. This condition is primarily associated with a specific metabolite of n-hexane [20].

Given the growing recognition of solvents that align with green chemistry principles, it is very desirable to replace traditional, potentially hazardous solvents with more environmentally benign alternatives. Notably, extraction efficiency has been found to exceed that of conventional solvents when these green solvents are used [21]. Among these, natural deep eutectic solvents (NADESs), a new generation of solvents based on natural compounds such as organic acids, amino acids, choline salts, and sugars [22], as well as biosynthetically more highly evolutionary metabolites, such as 1,8-cineole, p-cymene, D-limonene, menthol, menthone, thymol, and borneol [23,24] have attracted increasing attention.

NADESs are capable of dissolving a wide range of analytes with varying polarities, making them suitable for the extraction of numerous natural compounds [22]. Since the initial reports of NADESs in 2011, these solvents have been applied in the extraction of a variety of compounds, including chalcones, anthocyanins, phenolic acids, phenethyl alcohol, flavone lignans, biopolymers, zingerone, and xanthonoids [23]. Given that naphthoquinone pigments such as shikonin are susceptible to oxidative, thermal, and photodegradation, optimizing extraction conditions—such as solvent choice, extraction time, and temperature—is essential [1].

However, to our knowledge, the green extraction of shikonin derivatives remains underexplored. The only study addressing NADESs in this context reports the use of levulinic acid:glucose systems in ex situ hydroponic cultivation of *Alkanna tinctoria* using the nutrient film technique. This approach highlights the potential of integrating sustainable cultivation with NADES-based extraction without plant destruction [25]. Nevertheless, the direct application of NADESs for efficient extraction of shikonin derivatives from plant material remains largely unexplored.

The main hypothesis of this study is that VNADES-based systems may improve the recovery of shikonin derivatives compared to hexane due to enhanced solubilization via hydrogen bonding and other non-covalent interactions. This is in agreement with previous reports on NADES systems applied for the extraction of lipophilic natural products [21]. It was further assumed that extraction efficiency depend on the VNADES composition and molar ratio. It was also anticipated that differences in extract composition would be reflected in their biological activity. 

Based on these assumptions, this study aimed to develop and evaluate an innovative green chemistry-oriented extraction approach for shikonin derivatives from *Echium vulgare* L. roots using VNADESs. The study also aimed to compare the extraction efficiency, selectivity, and biological potential (assessed by cytotoxic, antioxidant, anti-inflammatory and antimicrobial tests) of this approach with that of conventional hexane extraction.

## 2. Results and Discussion

### 2.1. Identification and Determination of Shikonin Derivatives in Extracts of E. vulgare

#### 2.1.1. HPLC-PDA-ESI-TOF-MS Analysis

**Figure 1:**
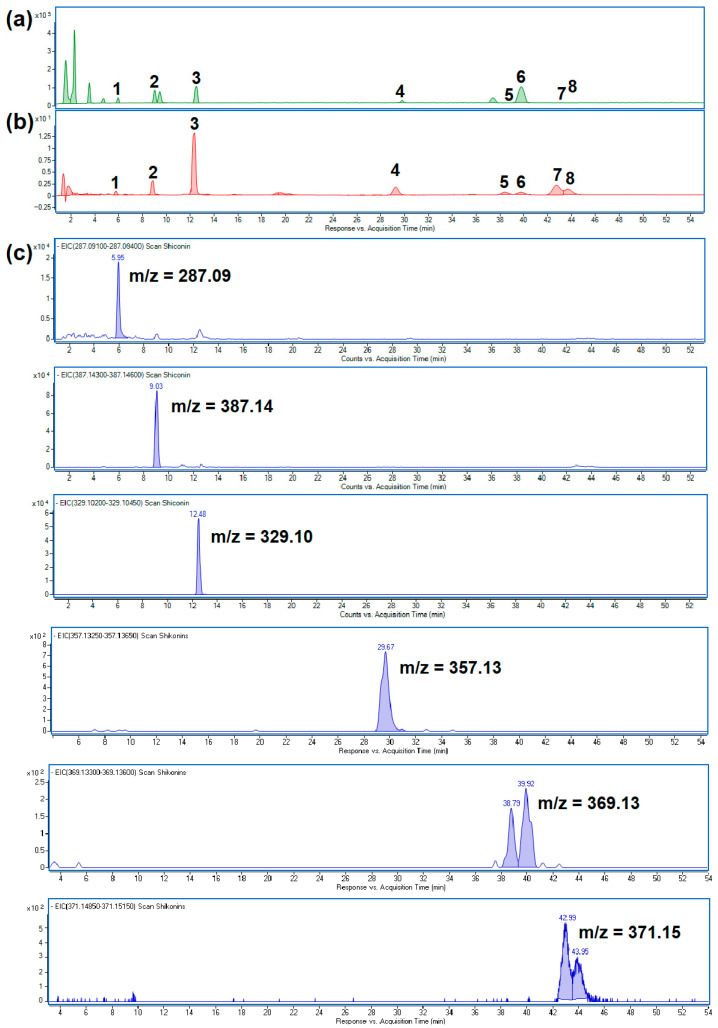
Chromatograms of *Echium vulgare* hexane extract: (**a**) HPLC-ESI-TOF-MS chromatogram; (**b**) HPLC-PDA chromatogram; (**c**) extracted ion chromatograms. The individual numbers of the peaks correspond to: 1—shikonin, 2—hydroxyisovalerylshikonin, 3—acetylshikonin, 4—isobutyrylshikonin, 5—dimetylacrylshikonin isomer 1, 6—dimetylacrylshikonin isomer 2, 7 isovalerylshikonin isomer 1, and 8—isovalerylshikonin isomer 2.

HPLC-PDA-ESI-TOF-MS analysis revealed the presence of shikonin and seven additional shikonin derivatives: hydroxyisovalerylshikonin, acetylshikonin, isobutyrylshikonin, dime-thylacrylshikonin isomers, or isovalerylshikonin isomers (Figure 1). However, data from Albreht et al. [26] suggests the presence of angelylshikonin instead of one of the dimethylacrylshikonin isomers and 2-methyl-n-butyrylshikonin instead of one of the isovalerylshikonin isomers. Albreht et al. [26] demonstrated the presence of these compounds in *Echium italicum* extract, confirming their structures using MS, as well as ^1^H and ^13^C NMR. It is worth noting that the chromatograms obtained by Albreht et al. indicate that these compounds have a similar retention time to the compounds identified in our study. In general, our chromatograms have a similar profile to those obtained by Albreht et al. [26]. However, as NMR analysis was not performed in our study, it was not possible to identify these metabolites with certainty. Therefore, it can be assumed that compounds 5–8 reported by us are shikonin derivatives with specific masses.

#### 2.1.2. HPLC-ESI-QTOF-MS/MS Analysis

**Figure 2:**
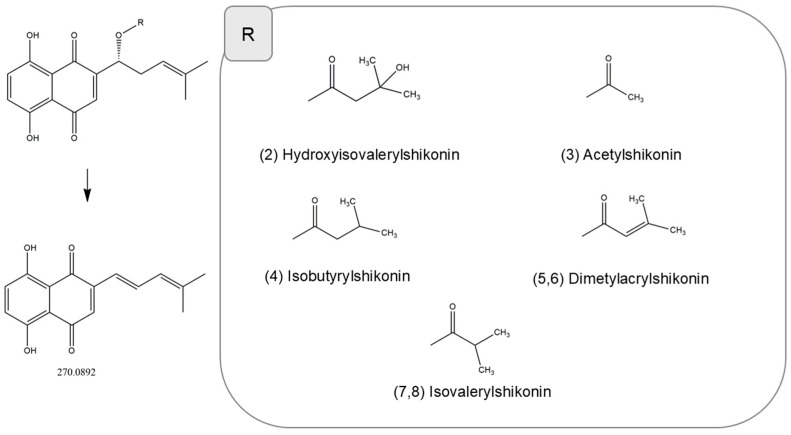
General structures of shikonin derivatives identified in a hexane extract from the roots of *Echium vulgare*, showing substituent variability and the proposed fragmentation pattern leading to the characteristic diagnostic ion (*m*/*z* 270).

To verify the identity of the compounds identified by HPLC-PDA-ESI-TOF-MS, an additional analysis was performed using HPLC-ESI-QTOF-MS/MS. Taking into account high-resolution monoisotopic mass measurements and the MS/MS fragmentation pattern, this analysis confirmed the presence of all eight compounds (Figure 2). These compounds have been reported by other authors [3,26,27]. The identities, retention times, observed [M-H]^−^ ions and product ions for individual components are presented in Table 1.

The MS/MS fragmentation patterns provided further structural confirmation of the identified shikonin derivatives. The *m*/*z* values of 237, 270 and 255 Da were registered among the MS/MS fragments, as presented in Table 1 and Figure S1. As shown in Figure 2, the general structure of shikonin derivatives along with their specific substituents is presented, together with a characteristic diagnostic product ion at *m*/*z*~270. This ion corresponds to the core shikonin moiety and was consistently observed in all analyzed shikonin derivatives. The formation of this fragment is in agreement with previous reports, where shikonin esters were found to share a common high-intensity fragment at *m*/*z* 270, presumably derived from the esterified shikonin core via fragmentation of the acyl substituent and/or subsequent CO elimination [26,27].

**Figure 3:**
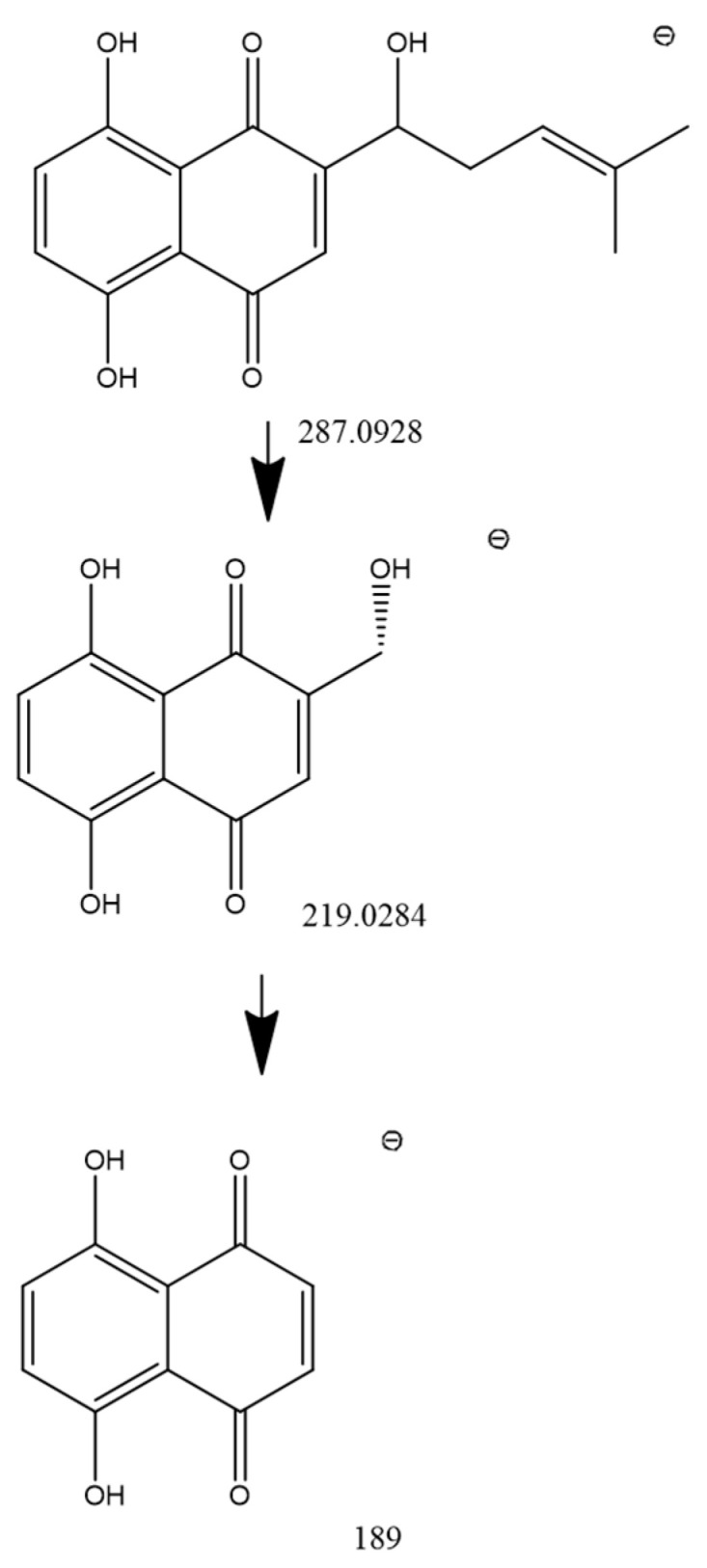
Proposed MS/MS fragmentation pattern of shikonin ([M-H]^−^) in negative ionization mode, including the [M-H]^−^ and major product ions at *m*/*z* 219.0284 and 189.

Interestingly, the characteristic fragment ion at *m*/*z* 270 was not observed for shikonin itself, suggesting a different fragmentation pattern compared to its ester derivatives. The proposed fragmentation scheme of shikonin is presented in Figure 3. This behavior may be attributed to the absence of acyl substituents, which limits the formation of a diagnostic fragment typically associated with esterified derivatives and favors alternative fragmentation routes. In negative ionization mode, these pathways may involve neutral losses such as C_5_H_8_/C_5_H_9_, CO, or H_2_O, as reported previously [28].

Additionally, a product ion at *m*/*z* ~255 was detected in all derivatives. This ion can be attributed to the formation of a core fragment, likely arising from cleavage of the acyl substituent and/or CO elimination [28].

Fragment ions at *m*/*z* 237 and 215 were consistently observed in several derivatives, including acetylshikonin, isobutyrylshikonin, dimethylacrylshikonin, and isovalerylshikonin, suggesting their origin from the fragmentation of the shikonin core. These ions likely arise from sequential fragmentation of the core ion. In particular, the ion at *m*/*z* 237 can be explained as a secondary product formed from *m*/*z* 255 via the neutral loss of H_2_O, consistent with previously reported CID-MS/MS fragmentation pathways [28].

The ion at *m*/*z* 215 may result from further fragmentation schemes involving additional neutral losses, such as H_2_O and CO, which are commonly observed in MS/MS spectra of organic compounds.

Furthermore, a characteristic product ion at *m*/*z* 117.0542 was observed exclusively for compound 2, corresponding to the hydroxyisovaleryl side chain, thus supporting its tentative identification as hydroxyisovalerylshikonin.

#### 2.1.3. Efficiency of Shikonin Extraction with VNADES Systems

Volatile natural deep eutectic solvents (VNADESs) have repeatedly proven to be effective in extracting plant metabolites, providing a viable alternative to toxic organic solvents such as hexane, acetone, and chloroform [29]. VNADESs have been shown to effectively extract isoquinoline alkaloids from *Chelidonium majus* [30] and metabolites from lichens [24,31]. The properties of NADESs depend heavily on the molar ratio of their constituent parts [29]. Therefore, different NADES compositions, including varying molar ratios of their constituents, should be considered during extraction optimization [24,31].

**Figure 4:**
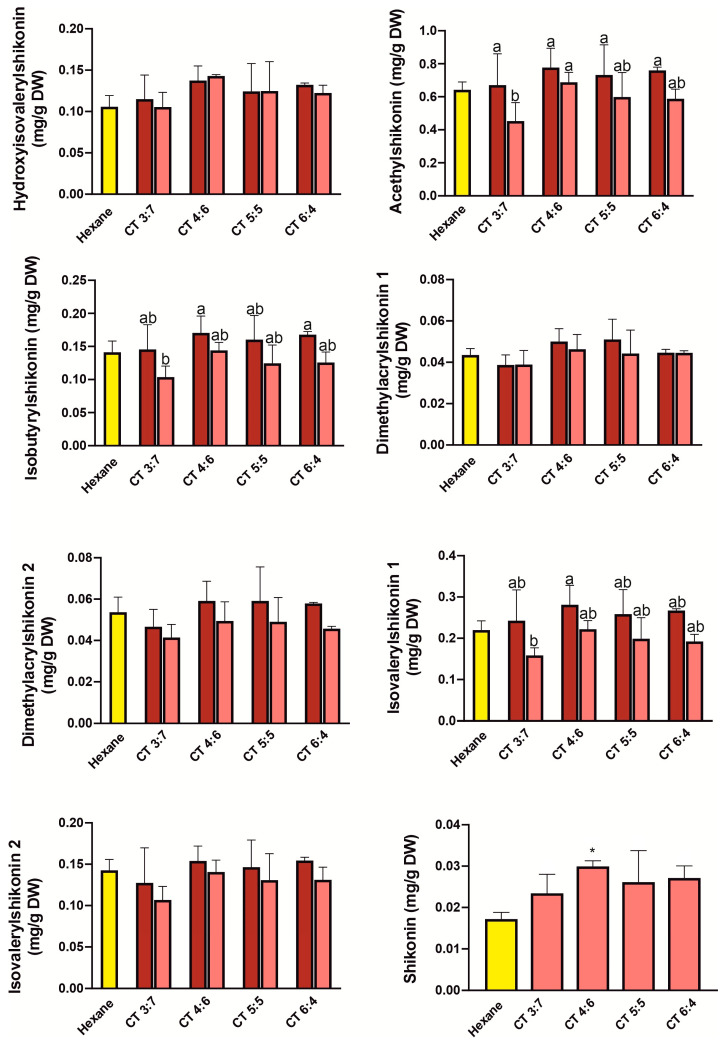
Yields of one-step extraction of shikonin derivatives from *Echium vulgate* roots with hexane and camphor:thymol (CT) systems. The red and pink bars represent the contents before and after freeze-drying, respectively. Yellow bars represent hexane. Data are expressed as mean ± SE (*n* = 5). Different letters above the bars indicate statistically significant differences determined by two-way ANOVA with replication (factors: molar CT, and freeze-drying treatment: before vs. after), followed by Tukey’s post hoc test for multiple comparisons. Additionally, Dunnett’s test was performed to compare each treatment with the control (hexane); statistically significant differences from the control are indicated by asterisks (* *p* < 0.05).

**Figure 5:**
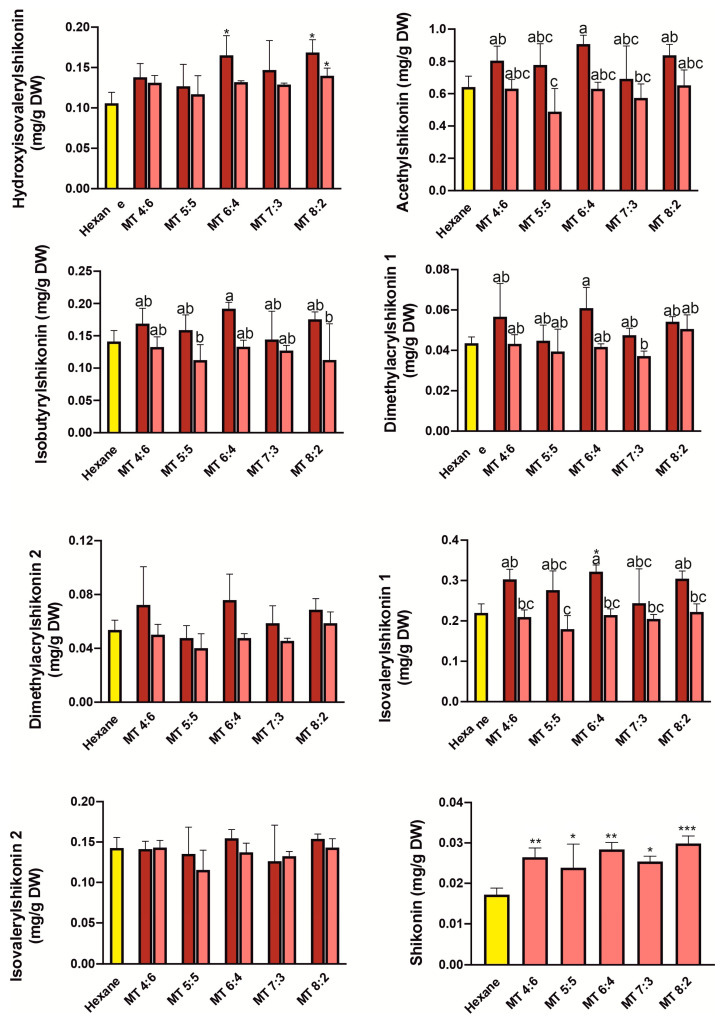
Yields of one-step extraction of shikonin derivatives from *Echium vulgate* roots with hexane and menthol:thymol (MT) systems. The red and pink bars represent the contents before and after freeze-drying, respectively. Yellow bars represent hexane. Data are expressed as mean ± SE (*n* = 5). Different letters above the bars indicate statistically significant differences determined by two-way ANOVA with replication (factors: molar CT, and freeze-drying treatment: before vs. after), followed by Tukey’s post hoc test for multiple comparisons. Additionally, Dunnett’s test was performed to compare each treatment with the control (hexane); statistically significant differences from the control are indicated by asterisks (* *p* < 0.05; ** *p* < 0.01; *** *p* < 0.001).

**Figure 6:**
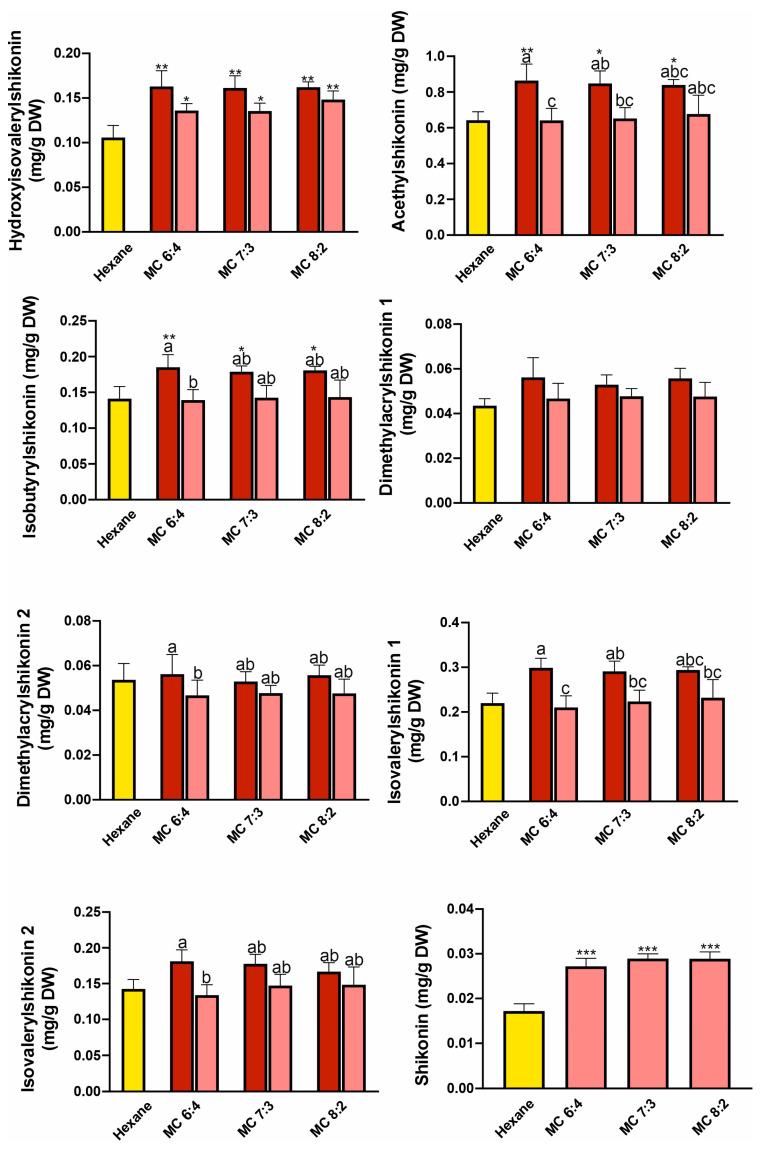
Yields of one-step extraction of shikonin derivatives from *Echium vulgate* roots with hexane and menthol:camphor (MC) systems. The red and pink bars represent the contents before and after freeze-drying, respectively. Yellow bars represent hexane. Data are expressed as mean ± SE (*n* = 5). Different letters above the bars indicate statistically significant differences determined by two-way ANOVA with replication (factors: molar CT, and freeze-drying treatment: before vs. after), followed by Tukey’s post hoc test for multiple comparisons. Additionally, Dunnett’s test was performed to compare each treatment with the control (hexane); statistically significant differences from the control are indicated by asterisks (* *p* < 0.05; ** *p* < 0.01; *** *p* < 0.001).

**Figure 7:**
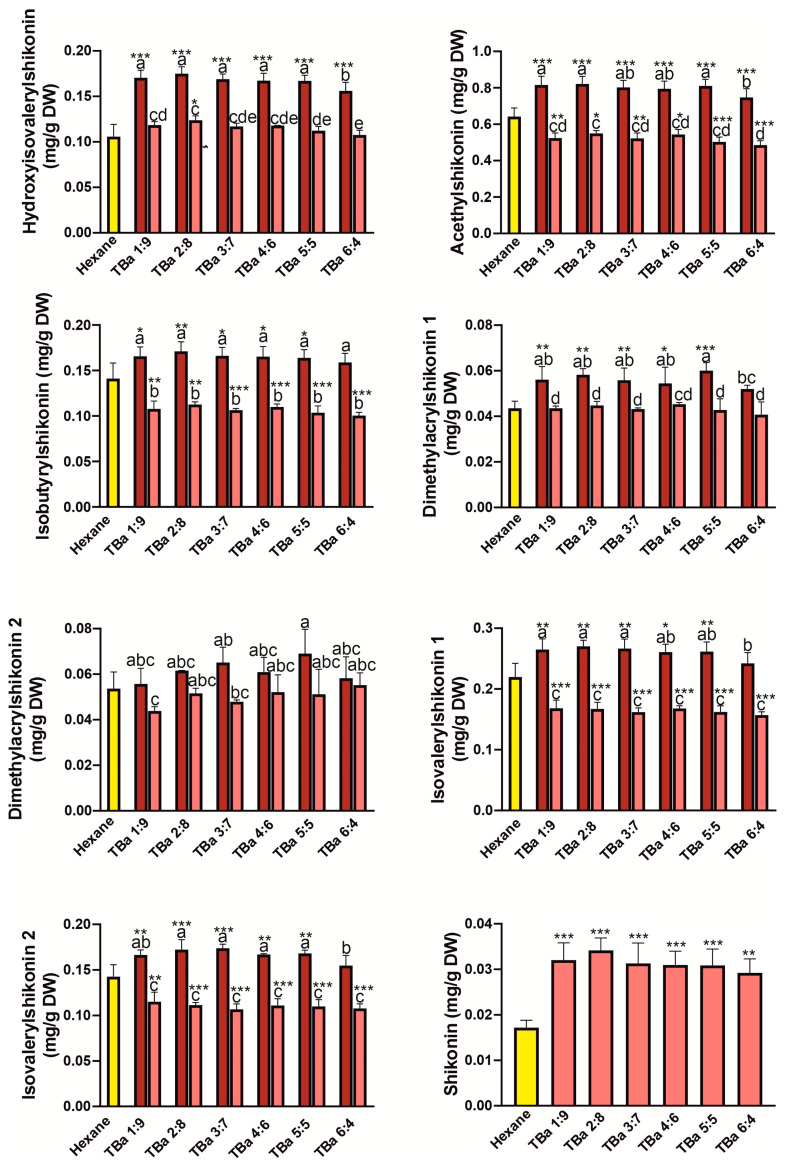
Yields of one-step extraction of shikonin derivatives from *Echium vulgate* roots with hexane and thymol:benzyl alcohol (TBa) systems. The red and pink bars represent the contents before and after freeze-drying, respectively. Yellow bars represent hexane. Data are expressed as mean ± SE (*n* = 5). Different letters above the bars indicate statistically significant differences determined by two-way ANOVA with replication (factors: molar CT, and freeze-drying treatment: before vs. after), followed by Tukey’s post hoc test for multiple comparisons. Additionally, Dunnett’s test was performed to compare each treatment with the control (hexane); statistically significant differences from the control are indicated by asterisks (* *p* < 0.05; ** *p* < 0.01; *** *p* < 0.001).

We compared the efficiency of single-step extractions using various NADES systems, which were: thymol: benzyl alcohol (TBa) 1:9—6:4, camphor: thymol (CT) 3:7–6:4, menthol: camphor (MC) 6:4–8:2, menthol: thymol (MT) 4:6–8:2 molar ratio, with the hexane extract, which is the most commonly used extractant for shikonin derivatives [18,26]. The results of single extraction with hexane and NADES systems (before and after freeze-drying of extracts) are presented in Figure 4, Figure 5, Figure 6 and Figure 7. Overall, all tested NADES crude extracts showed comparable or, in several cases, higher efficiency in extracting shikonin derivatives compared to hexane.

Slight exceptions were observed for the CT 3:7 (Figure 4) for dimethylacrylshikonin isomers, and for isovalerylshikonin isomer 2, as well as in the MT 5:5 ratio (Figure 5) for dimethylacrylshikonin 2 and isovalerylshikonin 2. Despite these minor differences, we observed that all four tested NADES systems were highly effective in extracting shikonin derivatives—particularly the TBa system (Figure 7). Among the tested systems, TBa showed the most consistent performance in shikonin extraction.

As shikonin co-eluted with the NADES components, it was not possible to determine its content in raw NADES extracts. Therefore, the shikonin content was determined after removal of NADESs by freeze-drying. Although lyophilization caused significant losses in the content of shikonin derivatives (especially in the TBa system), the shikonin content was higher than that in the hexane extract even when the NADES extracts were freeze-dried (Figure 4, Figure 5, Figure 6 and Figure 7). This may indicate that shikonin is less prone to loss due to evaporation under the tested lyophilization conditions than its derivatives.

In addition, for labile compounds such as shikonin derivatives, NADES systems may play a dual role as both extraction media and stabilizing environments. This effect has been attributed to the formation of extensive hydrogen-bonding networks between NADES components and solute molecules, which may influence molecular mobility and, consequently, the stability of labile compounds. Such effects have been suggested in previous studies on NADES systems [32].

The obtained results suggest that VNADES systems may represent a practical alternative to conventional organic solvents for the extraction of shikonin derivatives, reducing the reliance on volatile organic solvents. However, freeze-drying as a post-extraction step leads to substantial shikonin losses, representing a key limitation of this approach.

#### 2.1.4. Efficiency of Shikonin Extraction with Hexane

To further assess the potential of TBa 2:8 as a shikonin extractant, we compared exhaustive six-step extractions of *Echium vulgare* roots using both hexane (Table 2) and TBa 2:8 (Table 3). The efficiency of six-step extraction for other TBa systems is presented in the Supplementary Materials (Tables S2–S6).

The sum of extracted shikonin derivatives for TBa 2:8 crude extract did not include the amount of pure shikonin. Due to the co-elution between solvent matrix and shikonin, this compound was not determined in the analysis of crude NADES extracts. Despite this fact, the exhaustive extraction with TBa 2:8 yielded 2.032 mg/g dry weight roots, while the hexane-based extraction yielded 1.884 mg/g of total identified shikonins (in this case, a summary with pure shikonin). This indicates a slightly higher total yield for the TBa 2:8 system under the tested conditions. The six-step hexane extraction process revealed that steps one to three contributed most strongly to the extraction of individual shikonins and the dry weight of the extract residue (yield). The first step was found to isolate over 60% of the hexane-extractable compounds, including over 50% of shikonin, almost 65% of hydroxyisovalerylshikonin, and over 70% of the other shikonin derivatives. The next two steps yielded residues of 88%, extracting over 80% of shikonin and hydroxyisovalerylshikonin, over 95% of acetylshikonin, and all of the other analytes.

A slight deviation from the general trend observed in the fourth extraction step (Table 2) for hexane may be attributed to matrix-related effects, such as progressive removal of ballast compounds facilitating access to less accessible regions of the plant matrix. Interestingly, this behavior was not observed for TBa systems (Table 3 for TBa 2:8, and Tables S2–S6 for other TBa systems), suggesting a different extraction profile. While the exact mechanism remains to be elucidated, this phenomenon warrants further investigation.

### 2.2. Biological Panel

#### 2.2.1. Composition of the Dry Extracts Used for Estimating Biological Activity

Finally, we characterized the dry hexane and TBa 2:8 extracts obtained from single-step extractions, which were subsequently used in the biological assays (Table 4).

Although the TBa 2:8 provided a higher overall extraction yield in a single-step extraction (14.38 ± 1.13%) (Table 3), the dry hexane extract exhibited a higher concentration of shikonin derivatives, despite the lower overall yield (5.69 ± 0.91%) (Table 2). Higher extraction yield of TBa 2:8 extract may arise from the broader extraction capability of NADESs, which enables the co-extraction of both polar and non-polar constituents. In contrast, hexane, due to its strict non-polar nature, selectively extracts shikonins and other non-polar compounds. The ability of NADESs to extract compounds across a wide polarity range has been previously reported by Dai et al. [33].

The difference in extraction yields between tested solvents does not result from higher selectivity of hexane, but rather from the loss of compounds during lyophilization of the NADES extracts. The hexane extract was not lyophilized; instead, solvent removal was accomplished by allowing the extract to evaporate under a fume hood until complete dryness. To estimate the scale of these losses, we performed a stability test on hexane *Echium vulgare* extract (Table S1) to determine the stability of shikonin derivatives under lyophilization conditions. These additional tests revealed that the most abundant shikonin derivative, which is acethylshikonin, is also the most exposed to the losses during lyophilization: after 24 h, losses exceed approximately 80% of the amount, but after 72 h reach 88.6%. Losses after 72 h observed for other shikonins are: 80.9% for shikonin; 52.4% for beta-hydroxyisovalerylshikonin; 73.6% for isobutyrylshikonin; and 58.7%, 60.9%, 59.5%, and 56.3% for dimethylacrylshikonin 1 and 2, and isovalerylshikonin 1 and 2, respectively. Our observation corresponds with recent studies, which indicated that lyophilization caused losses in all five tested lichen metabolite contents, whereas in another evaporation method applying a rotary evaporator, it did not result in their loss. These results highlight the deep vacuum conditions (0.01 mbar) applied during freeze-drying as a direct factor contributing to these losses [31].

Other studies indicate that the extent of bioactive compound loss during lyophilization depends on the matrix of the lyophilized material. For example, one study reported losses of 84% for vitamin C in selected berries [34], whereas another investigation demonstrated only a 4% reduction in vitamin C content during evaporation of tomato purée [35]. On the other hand, scientists emphasized the fact that all shikonin derivatives are heat-, light-, pH- and oxygen-sensitive, which affects their stability and may increase their degradation [27,36].

Although freeze-drying is generally regarded as a mild dehydration method, it may still cause partial degradation of thermolabile or oxidation-sensitive compounds. Such degradation can result from oxidative and pH-related stresses occurring during freezing and secondary drying, when local temperature and oxygen exposure increase [37]. Taken together, these factors may partially account for the considerable losses of shikonin derivatives observed in the present study.

These findings underscore the importance of selecting an appropriate extraction medium based on the intended application. TBa 2:8 may be beneficial for maximizing total extract yield, while hexane enables direct recovery of shikonin-rich extracts by avoiding compound degradation during drying.

Our results also highlight a key limitation in the use of NADES systems for preparing dry extracts: significant degradation or loss of shikonin derivatives was observed during the lyophilization of all tested NADES extracts. Thus, while crude (non-lyophilized) NADES extracts—particularly TBa 2:8—contained higher levels of shikonins, the final concentration in the dry extract was lower compared to that of hexane. Accordingly, hexane can only be considered more selective toward shikonins when evaluating dried extracts.

These observations highlight a trade-off between extraction yield and selectivity, where NADES systems enable broader extraction of matrix components, while conventional non-polar solvents such as hexane provide more selective enrichment of shikonin derivatives in the final dry extract.

#### 2.2.2. Cytotoxicity Activity

A cytotoxicity test was performed on two cell lines: the human monocytic leukemia cell line (THP-1) and human dermal fibroblasts (HDFs). The THP-1 cell test, which involved differentiating the cells into macrophages using lipopolysaccharide (LPS), aimed to determine the concentration range for subsequent intracellular ROS and NO secretion inhibition tests (see Section 2.2.4). The HDF cytotoxicity study was designed to determine the safety profile of shikonin-containing extracts for skin use, given that *Echium* extracts are used in traditional medicine to treat various dermatological conditions.

**Figure 8:**
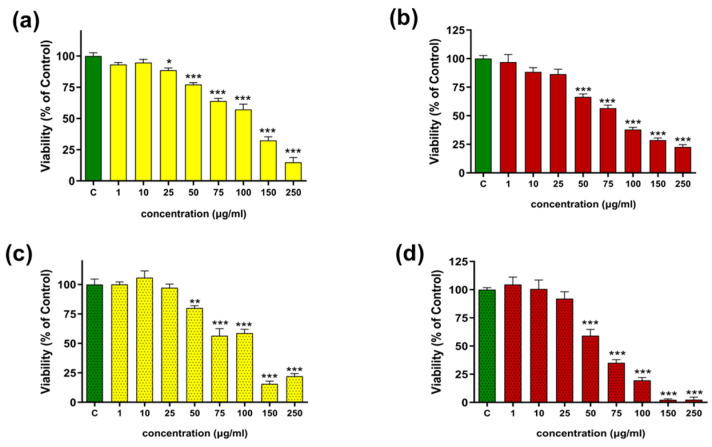
Effects of the hexane (bars of yellow) and T:Ba 2:8 (bars of red) extracts on: human monocytic leukemia cell line (THP-1) (**a**,**b**) and on LPS-stimulated macrophage cell (**c**,**d**) viability expressed as a percentage of the control (bar of green). Data are presented as mean ± SD (*n* = 3). Statistical analysis was performed using one-way ANOVA followed by Dunnett’s multiple comparisons test. Differences were considered statistically significant at *p* < 0.05 (*), *p* < 0.01(**), *p* < 0.001(***), relative to the control.

The tested extracts were shown to have no effect on THP-1 cell viability in the 1–25 µg/mL concentration range (see Figure 8a,b,d). However, a slight decrease in the survival of macrophages exposed to hexane extract at this concentration was observed (see Figure 8c). Exposure to higher concentrations of the extracts caused dose-dependent toxicity, which was evident at a concentration of 50 µg/mL.

**Figure 9:**
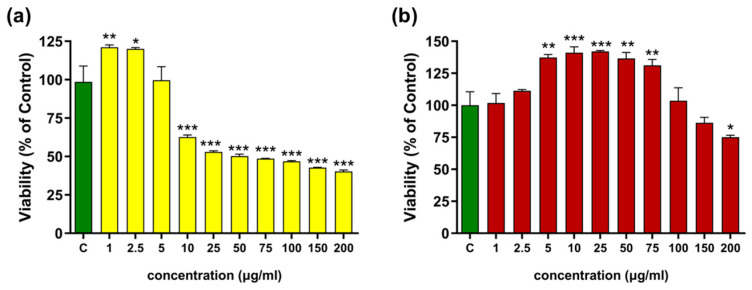
Assessment of resazurin reduction in fibroblasts (HDFs) for (**a**) hexane (bars in yellow) and (**b**) TBa (bars in red) extracts. Control cells (green bars) without the addition of test samples, for which viability was assumed to be 100%. Data are presented as mean ± SD (*n* = 3). Statistical analysis was performed using one-way ANOVA followed by Dunnett’s multiple comparisons test. Differences were considered statistically significant at *p* < 0.05 (*), *p* < 0.01(**), *p* < 0.001(***), relative to the control.

In HDFs, the hexane extract (Figure 9a) was well tolerated at the lowest doses. At 1–5 µg/mL, cell viability was slightly increased relative to the control (approximately 115–125% of the control, without statistically significant differences), indicating good tolerance of these concentrations. From 10 µg/mL, a clear and statistically significant reduction in viability was observed, down to ~60% of the control at 10 µg/mL and ~40–50% of the control in the 25–200 µg/mL range. The NADES-based extract in HDFs (Figure 9b) proved to be the best tolerated. At 5–75 µg/mL it induced a statistically significant increase in viability to about 130–140% of the control, indicating a pronounced stimulatory effect on cellular metabolic activity. At 100 µg/mL, viability approached control values, whereas at the highest concentrations (150–200 µg/mL) a moderate decrease was noted (to ~75–85% of the control), still without evidence of strong cytotoxicity. These findings indicate that the solvent used for extraction significantly influences the biological activity and cytotoxic profile of the obtained extracts. In the study by Akduman et al. [38], methanolic, acetonic, and aqueous root extracts of *Echium italicum* increased HDF cell viability, with statistically significant effects observed at concentrations of 250 µg/mL and higher for the first two extracts and for aqueous extract, at concentrations of 100 µg/mL.

In contrast, TBa-derived *Echium vulgare* extracts demonstrated a positive effect on HDF cell viability already at a markedly lower concentration of 5 µg/mL, suggesting higher biological activity at lower doses in the applied assay system. This suggests that the plant extract may positively influence fibroblast metabolic activity. This observation is consistent with previous reports indicating that shikonin can promote the proliferation of normal human cells, including fibroblasts and keratinocytes, supporting its potential role in tissue regeneration [39].

#### 2.2.3. Antioxidant Activity

**Figure 10:**
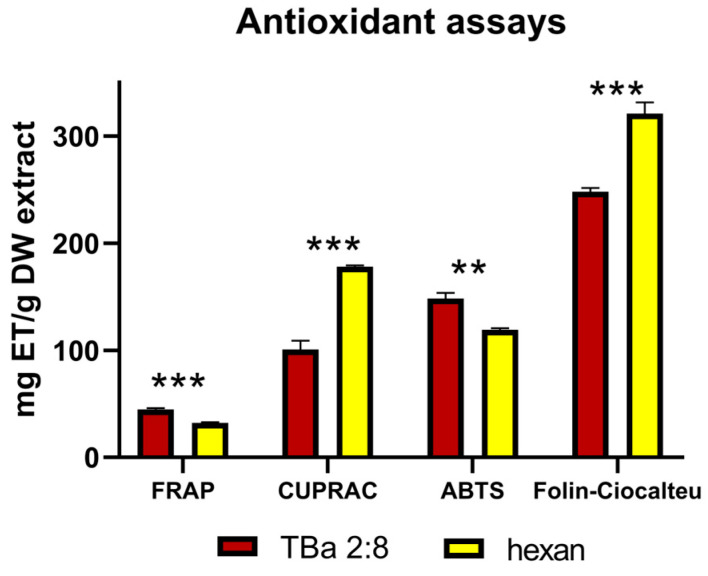
Antioxidant activity of the tested extracts, expressed as Trolox equivalent per gram of dry extract (mg ET/g DW extract). Data are mean ± SE (*n* = 5). Statistical differences between extracts obtained with the TBa 2:8 system and hexane were evaluated using Student’s *t*-test. Asterisks indicate statistically significant differences between the two extraction systems: *p* < 0.01 (**) and *p* < 0.001 (***).

In this study, the antioxidant activity of the extracts was evaluated using tests for the reduction of Fe^3+^ ions to Fe^2+^ (FRAP test), Cu^2+^ ions to Cu^+^ (CUPRAC test), activity against ABTS radicals, and the reduction of phosphotungstic and phosphomolybdic acids (Folin–Ciocalteu reaction). The results obtained are presented in Figure 10. In general, the observed activity depended on the type of test (lowest in the FRAP test and highest in the Folin–Ciocalteu reagent test). No extract showed higher activity in all tests: the hexane extract reduced copper ions and Folin–Ciocalteu reagent components more effectively than the TBa extract, but the opposite results were obtained in the FRAP and ABTS tests. The antioxidant potential of the hexane extract can be ranked as follows: Folin > CUPRAC > ABTS > FRAP, while for the TBa extract: Folin > ABTS > CUPRAC > FRAP.

It is difficult to determine whether the antioxidant activity of the tested extracts is high because the literature data on the activity of plant extracts varies widely depending on factors such as the plant species, type of raw material, and extractant used. For instance, Kozłowska et al. [40] demonstrated that extracts from eight herbal raw materials reduced ABTS radicals by between 30.035 and 896 mg ET/g and iron ions by between 17.52 and 455.5 mg ET/g in the FRAP test. In contrast, Walasek-Janusz et al. [41] demonstrated that the FRAP activity of extracts from *Sideritis scardica* and *Thymus vulgaris* was approximately 1.2 and 1.45 mg ET/g, respectively. Furthermore, scientists emphasize the fact that shikonin, alkannin and their derivatives may simultaneously exert antioxidant effects in tested oil substrates [42]. In another study, the authors demonstrated that an aqueous methanolic extract of *Lithospermum erythrorhizon* exerted significant antioxidant and cytoprotective effects in dermatological models, protecting human keratinocytes (HaCaTs) and neonatal dermal fibroblasts (HDF-ns) against H_2_O_2_- and UV-induced oxidative stress, limiting collagen degradation. These effects were attributed to the presence of phenolic constituents, including caffeic acid and shikonin derivatives [43].

Taken together, although the antioxidant response varied depending on the assay applied, the overall antioxidant potential of the hexane and TBa extracts can be considered comparable, as neither extract consistently outperformed the other across all four tests. Importantly, the dry TBa extract contained approximately threefold lower total shikonin content than the hexane extract, yet exhibited a similar antioxidant capacity. This observation suggests that the antioxidant activity observed in our study cannot be attributed solely to shikonins but may rather result from the combined or synergistic action of multiple extract constituents. This may also reflect differences in the chemical structure of the extracted compounds, as shikonin derivatives with different substituents may vary in their redox behavior and contribution to antioxidant activity.

Notably, although the TBa extract was less enriched in shikonins, it yielded a higher overall extraction efficiency, further supporting that this particular NADES exhibits a broader extraction capacity, enabling the simultaneous recovery of both polar and non-polar constituents that may collectively contribute to the antioxidant potential.

#### 2.2.4. Anti-Inflammatory Activity

**Figure 11:**
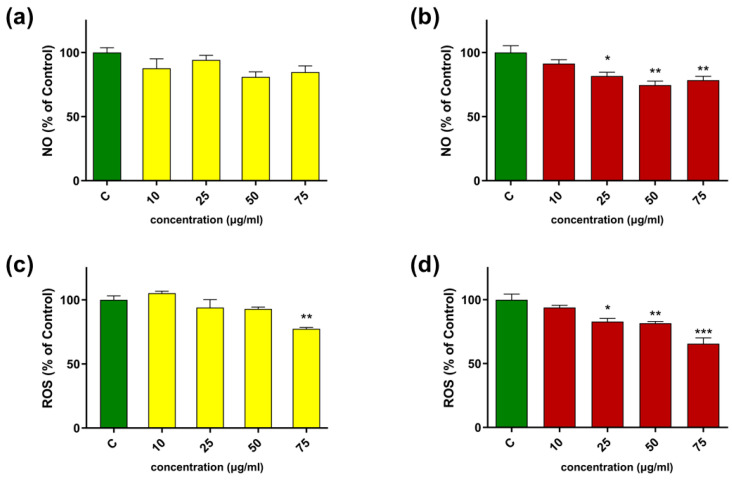
Influence of shikonin extracts, hexane (yellow bars) and TBa (red bars) on NO (**a**,**b**) and ROS (**c**,**d**) production in THP-1-derived macrophages. Control cells (green bars) were incubated in growing media only, for which NO or ROS level was assumed to be 100%. The average ROS and NO level represents the mean  ±  SD of three independent experiments (*n* = 3). Statistical analysis was performed using one-way ANOVA followed by Dunnett’s multiple comparisons test. Differences were considered statistically significant at *p* < 0.05 (*), *p* < 0.01(**), *p* < 0.001(***), relative to the control.

Notably, treatment with the TBa-extract reduced the production of intracellular ROS and NO at a concentration of 25 μg/mL (compared to the control), a level that was not toxic to either human monocytes or LPS-activated macrophages (see Figure 8). Higher concentrations of the extract did not significantly increase the dose-dependent inhibition of NO and ROS production. The hexane extract had no effect on NO production within the range of concentrations used (Figure 11a), while a significant reduction in oxidative stress was observed at a concentration of 75 µg/mL (Figure 11c). However, at this concentration, the extract reduced macrophage cell viability by approximately 60% (Figure 8c). Therefore, it can be concluded that, unlike the hexane extract, the TBa extract can reduce intracellular oxidative and nitrosative stress when used at non-toxic concentrations.

According to Kourounakis et al., the naphthazarin structure of shikonin and its derivatives contribute to the free radical scavenging properties, which may affect their anti-inflammatory activity [44]. Notably, the ROS-generating capacity of shikonin is considered a key mechanism underlying its well-documented anti-cancer activity [45]. At the same time, shikonin has been reported to exhibit stronger COX inhibitory potency than alkannin, but also higher cytotoxicity and pro-oxidant activity [46]. These findings suggest that the biological effects of shikonin derivatives may depend on their relative abundance and redox behavior. In this context, the slightly lower cytotoxicity observed for the NADES extract in our study may be related to its lower content of shikonin derivatives.

The anti-inflammatory activity of shikonin and its derivatives has been demonstrated in several in vivo and in vitro models. In carrageenan-induced paw edema, selected derivatives such as *β,β*-dimethylacrylshikonin, isovalerylshikonin, and acetylshikonin exhibited pronounced anti-inflammatory effects. Moreover, the *n*-hexane extract obtained from the roots of *L. erythrorhizon*, which contained these compounds that were identified as the most relevant, were shown to suppress TNF-α promoter activity in vivo, with shikonin and isobutyrylshikonin displaying the strongest inhibitory effects. Additional studies confirmed that shikonin attenuates key inflammatory pathways, including NF-κB and ERK signaling, leading to reduced expression of COX-2, iNOS, nitric oxide, and prostaglandin E_2_ production in activated macrophages [47].

#### 2.2.5. Antimicrobial Activity

The antimicrobial activity of hexane and NADES-based (TBa 2:8) extracts was evaluated by determining their minimum inhibitory concentration (MIC) and minimum bactericidal concentration (MBC) against a panel of Gram-positive, Gram-negative, and fungal strains. The results are shown in Table 5. Gram-positive bacteria were generally the most sensitive to the extracts, particularly the *S. aureus* and *S. epidermidis* strains (MIC = 0.004 mg/mL, MBC = 0.008 mg/mL for the hexane extract), as well as *Micrococcus luteus* and *Bacillus subtilis* (MIC = 0.002 mg/mL, MBC = 0.004 mg/mL for the hexane extract), and Bacillus cereus ATCC 10876 (MIC = 0.008 mg/mL, MBC = 0.03 mg/mL for the hexane extract).

Ethanolic extracts of *E. vulgare* have been reported to inhibit *Staphylococcus epidermidis* in disc diffusion assays, and in our study, *S. epidermidis* was also among the more susceptible Gram-positive strains tested with the same extracts, highlighting its relative sensitivity compared to other strains. The choice of solvent strongly influences the extraction of bioactive compounds, as highlighted by previous studies reporting that methanolic extracts of *E. vulgare* exhibited no detectable antibacterial activity [48]. Activity of different polarity aboveground *Echium vulgare* extracts against *Candida albicans* has been also evaluated by Bošković et al. The study indicates that the extracts of *Echium vulgare* L. exhibited a certain degree of antimicrobial activity, while chloroform and ethanol extracts exhibited the best antimicrobial activity compared to the other (ethyl acetate, acetone, and petroleum) tested extracts [49].

The MBC/MIC ratio values were below 4 in most cases, indicating that both the hexane extract and the TBa extract exhibited bactericidal activity [50]. Bacteriostatic activity was only observed for *Bacillus cereus* ATCC 13061 (hexane extract), and for *S. aureus* ATCC 6538 and *S. aureus* ATCC43300 (TBa extract).

In contrast, Gram-negative bacteria exhibited markedly lower susceptibility. The MIC values for Gram-negative bacteria were almost always 2 mg/mL for the hexane extract and 4 mg/mL for the TBa 2:8 extract. *Salmonella* Typhimurium ATCC 14028 was the only strain that was more sensitive to the tested extracts (MIC = 0.5 and 2 mg/mL for the hexane and TBa extracts, respectively). The reduced susceptibility of Gram-negative bacteria can be attributed to the presence of an outer membrane rich in lipopolysaccharides, which limits the permeability of hydrophobic compounds. In addition, previous studies have suggested that certain Gram-negative species may metabolize plant-derived naphthoquinones, including shikonin, potentially contributing to reduced activity [51].

In terms of antifungal activity, the hexane extract was two to four times more active than the TBa extract. The MIC values for the hexane extract were found to range from 0.25 to 0.5 mg/mL for *Candida* strains, and from 0.125 mg/mL for *Geotrichum candidum*. The TBa extract inhibited the growth of all the fungi tested in a concentration range of 1–2 mg/mL. The MFC values were generally two to four times higher than the MIC values and were only equal to the MIC values in a few cases (hexane extract: *C. glabrata* ATCC 15126, *C. albicans* ATCC 14053; TBa extract: *C. parapsilosis* ATCC 22019, *C. glabrata* ATCC 66032). All the tested strains showed fungicidal activity (MFC/MIC ≤ 4).

Sasaki et al. compared the antifungal effects of shikonin with the standard antifungal agent fluconazole and demonstrated that the fungicidal activity of shikonin was four times higher against *Candida krusei* and comparable to fluconazole against *C. glabrata*. Deoxyshikonin also exhibited greater activity against *C. krusei* than fluconazole. Although other derivatives (acetylshikonin, β-hydroxyisovalerylshikonin) were less active than the reference drug, all tested compounds showed measurable antifungal effects. In this study, *C. albicans* appeared to be more resistant to shikonin derivatives than to fluconazole [52]. However, a subsequent study reported that shikonin exhibited markedly stronger activity against certain fluconazole-resistant *C. albicans* strains, with its antifungal effect being more than 16-fold greater than that of fluconazole [53].

Overall, the antimicrobial profiles obtained in this study are in agreement with the known bioactivity of shikonin-rich extracts [10,54,55,56], supporting the role of solvent selection in modulating the chemical composition and biological potency of the final extract. VNADES systems can therefore serve as green extraction media for shikonin derivatives; however, post-extraction processing, particularly freeze-drying, remains a critical factor affecting final yield and bioactivity.

## 3. Materials and Methods

### 3.1. Reference Standards, Chemicals and Plant Material

Shikonin (≥98%), DL-menthol (M ≥ 95%), (±)-camphor (C ≥ 95.5%), thymol (T ≥ 99%), benzyl alcohol (Ba ≥ 99%), methanol (LC-MS), acetonitrile (LC-MS), hexane (≥99%), trifluoroacetic acid (HPLC ≥ 99%), formic acid (98–100% for LC-MS LiChropur™, Merck, Darmstadt, Germany), 2,2′-azinobis-(3-ethylbenzthiazoline-6-sulfonic acid) (ABTS 98%), potassium persulfate (≥99%), 1,3,5-Tri(2-pyridyl)-2,4,6-triazine (TPTZ), iron (III) chloride (≥98%, FeCl_3_), sodium acetate (≥99%), sodium carbonate (Na_2_CO_3_ ≥ 99.5%), Folin–Ciocalteu reagent and 6-hydroxy-2,5,7,8-tetramethylchroman-2-carboxylic acid (Trolox) were purchased from Sigma-Aldrich (St. Louis, MO, USA). Acetic acid (99.5–99.9%) and hydrochloric acid (36%) were supplied by Avantor Performance Materials Poland S.A. (Gliwice, Poland). Water was deionized and purified using ULTRAPURE Millipore Direct-Q^®^ 3UV-R (Merck, Darmstadt, Germany). Dulbecco’s Modification of Eagle’s Medium (DMEM) was purchased from VWR International (Radnor, PA, USA). Antibiotics (100 U/mL penicillin and 1000 μg/mL streptomycin), Fetal Bovine Serum (FBS), and phosphate-buffered saline (PBS; pH 7.00 ± 0.05) were purchased from Genos (Łódź, Poland). Resazurin sodium salt (RES) Sigma-Aldrich (Poznań, Poland), HRP conjugate (horseradish peroxidase), Stop Solution (sulfuric acid solution), and 3,3′,5,5′-tetramethylbenzidine (TMB) were purchased from Elabscience (Houston, TX, USA), while RIPA buffer was obtained from EURx (Gdansk, Poland).

*Echium vulgare* plants were collected in Lublin in May 2024. The roots were separated from the above-ground parts, washed in distilled water, pre-dried with blotting paper, and then dried naturally. Samples of the plant material were deposited at the Department of Analytical Chemistry at the Medical University of Lublin.

### 3.2. Identification and Determination of Shikonin Derivatives in Extracts of E. vulgare

Identification of shikonin derivatives was carried out in two independent laboratories (Department of Pharmacognosy with Medicinal Plants Garden and Department of Analytical Chemistry, Medical University of Lublin) using two different LC-MS instruments.

#### 3.2.1. HPLC-PDA-ESI-TOF-MS Identification of Shikonin Derivatives

The mass spectrometry data were recorded using an Infinity Series II ultra-high-performance liquid chromatography (UHPLC) system coupled with an Agilent 6224 ESI/TOF mass spectrometer (Agilent Technologies, Santa Clara, CA, USA). The chromatographic system utilized a Kinetex C18 reversed-phase column 100 Å (150 mm × 2.1 mm; 1.7 μm particle size) (Phenomenex, Torrance, CA, USA). The column thermostat was maintained at 30 °C and the flow rate was set to 0.2 mL/min. Isocratic elution was performed using a mobile phase consisting of 40% acetonitrile acidified by formic acid (0.05%).

Chromatographic analysis was performed within the wavelength range from 200 to 600 nm. The MS parameters were as follows: drying gas temperature of 325 °C, flow rate of 8 L/min, nebulizer pressure of 30 psi, capillary voltage of 3500 V, fragmentor voltage of 180 V, and skimmer voltage of 65 V.

#### 3.2.2. HPLC-ESI-QTOF-MS/MS Identification of Shikonin Derivatives

The experiment was conducted using an instrument consisting of a degasser, a binary pump for the solvent, a peristaltic pump for reference ions, an autosampler, a UV detector, and a Q-TOF detector with an electrospray ionization source. For the analysis, the Agilent Technologies (Santa Clara, CA, USA) HPLC-ESI-Q-TOF-MS platform was used, which included an HPLC chromatograph (1200 series) with a Zorbax Eclipse Plus RP-18 chromatographic column (150 mm × 2.1 mm; 3.5 µm particle size). The system consisted of a degasser (G1322A), a binary pump (G1312C), an autosampler (G1329B), a photodiode array detector—DAD (G1315D), and a mass spectrometer (G6530B). For MS spectral acquisition and data processing, Agilent MassHunter workstation software (version B.10.00) was used. The following gradient of solvent A: water with 0.1% formic acid and solvent B: acetonitrile with 0.1% formic acid was applied: 0–2 min 30% B, 2–8 min with 30% B transitioning to 60% B, 8–17 min with 60% B transitioning to 95% B, 17–22 min 95% B, 22–25 min with 95% B transitioning to 1% B. The analysis lasted 30 min, the injection volume was set at 10 µL, the flow rate at 0.2 mL/min, and the temperature at 20 °C. The identification of compounds was based on high-resolution mass measurements, MS/MS fragmentation patterns, and consideration of previously published data regarding the composition of this specific plant species using the following mass spectrometer settings: the *m*/*z* range of 100–1400 Da, the gas and sheath gas temperatures of 250 and 300 °C, the gas flows of 12 L/min, the fragmentor voltage of 110 V, the skimmer voltage of 65 V, the capillary voltage of 3000 V, and the collision energies of 10 and 20 V.

#### 3.2.3. The HPLC-PDA Determination of Shikonin Derivatives

The shikonin derivatives were analyzed using an EliteLaChrom chromatograph equipped with a UV-VIS PDA detector (Hitachi, Tokyo, Japan) and EZChrom Elite software (version 3.3.2 SP2 build 3.3.2.1037) from Merck(Darmstadt, Germany). A reversed-phase core-shell column (Kinetex, Phenomenex, Aschaffenburg, Germany) was used at a temperature of 35 °C. The column had the following specifications: 25 cm × 4.6 mm internal diameter and 5 μm particle size. Isocratic elution was performed using a mobile phase consisting of 55% acetonitrile acidified by trifluoroacetic acid (0.1%), with a flow rate of 1 mL/min. The compounds in the plant extracts were identified based on their UV-Vis spectra (λ = 190–600 nm). Quantitative analysis was performed at λ = 277 nm. The data were recalculated based on the standard curve prepared for the shikonin standard and are expressed per gram of plant dry weight (DW).

### 3.3. Exhaustive Extraction

Approximately 500 mg of dried, ground *E. vulgare* root was placed in a 5 mL Eppendorf tube and extracted with 5 mL of hexane. The sample was sonicated in an ultrasonic water bath for 15 min at ambient temperature (ultrasound frequency: 35 kHz; Sonorex RK 512 H, Bandelin, Berlin, Germany). Following sonication, the samples were centrifuged at 4414× *g* for 15 min at 20 °C. The resulting supernatant was transferred to a pre-weighed 5 mL volumetric flask and left until complete evaporation of the solvent, yielding the dry extract. The plant material remaining in the Eppendorf tube was then treated with a fresh 5 mL portion of hexane, and the process of sonication, centrifugation, and transfer of the supernatant to the next volumetric flask was repeated. This cycle was conducted six times to ensure exhaustive extraction.

Under the same conditions, the extraction was also performed using thymol:benzyl alcohol in molar ratio 2:8 (TBa 2:8).

The extraction yield at each stage was calculated based on the mass difference between the flask containing the dry extract and the empty pre-weighed flask. The yield was expressed as milligrams of dry residue per gram of raw plant material.

Each flask containing the dry residue was subsequently made up to 5 mL with methanol. The methanol-dissolved dried hexane extracts were then analyzed by HPLC-DAD to quantify the content of shikonin derivatives at each extraction step as well as for qualitative analysis by HPLC-MS. All experiments were performed in triplicate.

### 3.4. Preparation of VNADESs

All volatile NADESs were obtained by heating the mixed components in a water bath without additional mixing for 30 min at 60 °C. The selection of VNADES components (thymol, benzyl alcohol, menthol, and camphor) was based on our previous study, which demonstrated their high potential for dissolving lipophilic natural products and forming stable eutectic systems [21]. This selection was further supported by their physicochemical properties, including high lipophilicity, hydrogen-bonding and π-π interaction potential, as well as their suitability to form low-melting mixtures. Their volatility was also considered advantageous for potential removal, and preliminary experiments confirmed their high extraction efficiency.

Liquids were made in the following systems and molar ratios: thymol:benzyl alcohol (TBa 1:9–6:4); camphor:thymol (CT 3:7–6:4); menthol:camphor (MC 6:4–8:2); and menthol:thymol (MT 4:6–8:2).

### 3.5. Optimization of Extraction Using VNADESs

A single-step extraction was applied at this stage to enable rapid screening of different VNADES systems and comparison of their extraction efficiency. Approximately 200 mg of dried and ground *E. vulgare* root was transferred into a 5 mL Eppendorf tube and extracted using 2 mL of a NADES system (extractions were performed with all prepared NADES systems (CT; MT; MC; and TBa)). The extraction procedure involved sonication and centrifugation under the same conditions previously described for the exhaustive hexane extraction. Following centrifugation, the supernatant was transferred into a vial, and diluted with an equal volume of methanol. Each extract sample was prepared and analyzed in triplicate. Quantitative analysis of the extracts was performed by HPLC-PDA according to the method described in Section 2.2.3.

### 3.6. Evaporation of VNADES-Based Extracts

A 0.5 mL sample of the extracts was freeze-dried at 0.01 mBar for 72 h (Alpha 2–4 LDplus Freeze Dryer; Martin Christ, Osterode am Harz, Germany). The dry residues were then weighed, dissolved in MeOH, and HPLC-PDA analyzed according to the method described in Section 3.2.3., to determine the loss of shikonins during the freeze-drying process.

For biological activity tests, the lyophilized TBa 2:8 extract and the dry hexane extract were used. The conditions for extraction are described in the section ‘Exhaustive Hexane Extraction’. A liquid-to-solid (L:S) ratio of 1 mL per 100 mg was maintained, and a single extraction was performed.

### 3.7. Cytotoxicity Evaluation

As a model of human macrophages, peripheral blood acute monocytic leukemia cells, THP-1s, were used (TIB-202; ATCC, Manassas, VA, USA). THP-1 cells were cultured in RPMI-1640 containing 10% FBS. Both media were also supplemented with antibiotics (100 U/mL penicillin and 1000 μg/mL streptomycin; Thermo Fisher Scientific, Waltham, MA, USA). To activate the THP-1 cells into macrophages, cells were incubated for 48 h (4 × 10^5^ cells/well of a 24-well plate) in the presence of 100 ng/mL of Phorbol 12-myristate 13-acetate (PMA; Sigma-Aldrich). Differentiated adherent cells were washed with culture medium and incubated for another 24 h in fresh culture media without PMA to obtain resting macrophages (M0). Cells were grown at 37 °C in a humidified incubator set to 5% CO_2_ and 95% air.

Human dermal fibroblasts (HDFs) were obtained from CLS Cell Lines Service (Eppelheim, Germany) and were maintained in Dulbecco’s Modified Eagle’s Medium (DMEM; Biological Industries, Cromwell, CO, USA) supplemented with sodium pyruvate, L-glutamine, glucose (4.5 g/L), and 10% fetal bovine serum (FBS; Genos, Łódź, Poland). To limit microbial contamination, medium was further supplemented with antibiotics (1% *v*/*v*; final concentrations: 100 U/mL penicillin and 1000 μg/mL streptomycin; Thermo Fisher Scientific, Waltham, MA, USA). Cells were grown in 75 cm^2^ culture flasks (Googlab Scientific, Rokocin, Poland) at 37 °C in a humidified incubator set to 5% CO_2_ and 95% air. Adherent cultures were passaged by trypsinization upon reaching ~70–80% confluence. Cytotoxicity of the shikonin extracts was determined using the resazurin reduction (Alamar Blue) assay (Merck KGaA, Darmstadt, Germany), following Ziemlewska et al. [57] with minor modifications. Cells were seeded onto black, flat-bottom 96-well plates (Googlab Scientific, Rokocin, Poland). After a 24 h conditioning period, cultures were exposed to the test extracts (1–200 μg/mL) for 24 h. Control wells containing untreated HDF cells maintained in their complete medium. Following exposure, the test solutions were aspirated and replaced with 60 μM resazurin. After a 2 h incubation, fluorescence was recorded at λ = 570 nm using a microplate reader (Thermo Fisher Scientific, Waltham, MA, USA). Cell viability was expressed relative to untreated controls, defined as 100%. Each concentration was tested in triplicate in three independent experiments.

### 3.8. Antioxidant Panel

Antioxidant activity was assessed using three tests: ABTS, FRAP and Folin–Ciocalteau reagent. Tests were conducted in accordance with previously published procedures: Gębalski et al. [58], Soja et al. [59] and Stasińska-Jakubas et al. [60] for ABTS, FRAP and Folin–Ciocalteu reagent, respectively. Values were expressed as the equivalent of mg of Trolox per gram of dry extract.

### 3.9. Anti-Inflammatory Panel

To measure the intracellular ROS or NO level, THP-1-derived macrophages growing on a 24-well plate were cultured for 24 h in presence of tested extracts or culture medium alone (control). To access ROS levels, cells were incubated with 2′,7′-dichlorodihydrofluorescein diacetate (H2 DCF-DA, Sigma, USA) for 60 min at 37 °C in the presence of 5% CO_2_, whereas to measure intracellular NO levels, cells were incubated with 4-amino-metyl-2′,7′-difluorofluorescein diacetate (DAF-FM DA; Sigma, USA). The intensity of fluorescence was measured in at least 10 000 events per sample using a FACSCalibur™ flow cytometer (BD Biosciences, San Jose, CA, USA) and the results were analyzed using FlowJo software (Version v10.10, FlowJo LLC, Ashland, OR, USA).

### 3.10. Antimicrobial Activity Panel

The antimicrobial profile of the extracts was evaluated in a panel of microorganisms from the American Type Culture Collection (ATCC, Manassas, VA, USA), and the Centers for Disease Control and Prevention (CDC, Atlanta, GA, USA) including Gram-positive bacteria: *Staphylococcus aureus* ATCC 25923, *S. aureus* ATCC 6538, *S. aureus* ATCC 29213, *S. aureus* ATCC BAA 1707, *S. aureus* ATCC 43300, *Staphylococcus epidermidis* ATCC 12228, *Enterococcus faecalis* ATCC 29212, *E. faecalis* ATCC 51299, *Enterococcus faecium* ATCC 19434, *Micrococcus luteus* ATCC 10240, *Bacillus subtilis* ATCC 6633, *Bacillus cereus* ATCC 13061, and *B. cereus* ATCC 10876; Gram-negative bacteria: *Salmonella enteritidis* ATCC 13076, *Salmonella* Typhimurium ATCC 14028, *Proteus mirabilis* ATCC 12453, *Bordetella bronchiseptica* ATCC 4617, *Escherichia coli* ATCC 25922, *E. coli* ATCC 35218, *Klebsiella pneumoniae* ATCC 13883, *K. pneumoniae* ATCC BAA 2146, *Enterobacter aerogenus* ATCC 13048, *Pseudomonas aeruginosa* ATCC 27853, *p. aeruginosa* NIL, and *Acinetobacter baumanii* ATCC 19606; *Candidia* yeasts: *C. albicans* ATCC 10231, *C. albicans* ATCC 2091, *C. albicans* ATCC 14053, *C. auris* CDC B11903, *C. glabrata* ATCC 15126, *C. glabrata* ATCC 90030, *C. glabrata* ATCC 66032, *C. parapsilosis* ATCC 22019, *C. lusitaniae* ATCC 3449, *C. tropicalis* ATCC 1369, and *Candida krusei* ATCC 14243; and *Geotrichum candidum* ATCC 34614. The strains were provided by the local collection of the Department of Pharmaceutical Microbiology, Medical University in Lublin. The study followed a previously published procedure [11] and according to the European Committee on Antimicrobial Susceptibility Testing (EUCAST) guidelines [61]: microbial suspensions were prepared in sterile saline (0.85% NaCl) with an optical density of 0.5 McFarland standard—1.5 × 108 colony-forming units (CFUs) per mL. Mueller–Hinton medium (Biomaxima, Lublin, Poland) was used with a series of 2-fold dilutions of the tested substances in a range of final concentrations from 80 to 0.08 mg/mL. The antibacterial activity of extracts was screened on the basis of the minimal inhibitory concentration (MIC). It was determined with the broth microdilution method, based on the European Committee on Antimicrobial Susceptibility Testing (EUCAST) guidelines [61]. MBC (minimal bactericidal concentration) and MFC (minimal fungicidal concentration) were estimated with the broth microdilution technique by plating out the contents of wells that showed no visible growth of bacteria onto Mueller–Hinton agar and incubating at 35 °C for 18 h. The MIC, MBC, and MFC values were given in mg/mL in accordance with the EUCAST references [61].

### 3.11. Statistical Analysis

Extraction experiments and antioxidant activity assays were performed in five independent replicates (*n* = 5), whereas anti-inflammatory activity assays conducted on macrophages were performed in three independent replicates (*n* = 3). Results are presented as mean ± standard deviation (SD). Statistical analyses were performed using GraphPad Prism 8 (GraphPad Software, USA). For extraction experiments, the effects of the extraction system and freeze-drying treatment were evaluated using two-way ANOVA with replication, followed by Tukey’s post hoc test for multiple comparisons. Additionally, Dunnett’s test was used to compare each treatment with the control sample (hexane). For antioxidant activity assays, statistical differences between extraction systems were evaluated using Student’s *t*-test. Differences were considered statistically significant at *p* < 0.05.

## 4. Conclusions

This study confirmed the effectiveness of VNADESs as a green solvent for extracting shikonins from *E. vulgare* roots. The VNADES systems provide comparable or higher levels of shikonin extraction than hexane. However, the overall performance of VNADESs is strongly influenced by post-extraction processing, as freeze-drying the extracts resulted in significant losses of shikonins.

The biological activity of the obtained extracts was closely linked to their chemical composition. The hexane extract, enriched in shikonin derivatives, exhibited higher antimicrobial activity, whereas the TBa 2:8 extract showed lower cytotoxicity and distinct anti-inflammatory effects, including ROS and NO inhibition not observed for the hexane extract. These results indicate a clear trade-off between extraction selectivity and biological profile.

A key limitation of VNADES systems is associated with the removal of the extraction medium, which leads to partial loss of target compounds and may affect the final bioactivity. This highlights the need for improved separation strategies. Potential solutions include optimization of freeze-drying conditions, application of non-lyophilized extract formulations, or alternative purification approaches such as liquid–liquid extraction with biocompatible solvents or solid-supported systems.

From a practical perspective, VNADESs represent a promising platform for green extraction on a laboratory scale. Their use of naturally derived components, low toxicity, and variable composition support their applicability in sustainable extraction strategies. In addition, recyclability and reuse of deep eutectic solvent systems have been proposed in related studies, where solvent recovery and reuse were shown to contribute to reduced process costs and improved sustainability although systematic investigations for VNADESs specifically remain limited.

## Figures and Tables

**Table 1:** The list of tentatively identified metabolites present in the investigated samples (DBE—double bond equivalent—the number of double bonds and rings, Error—error of *m*/*z* measurement, Rt—retention time).

No	Ion Type	Ionization [+/-]	Molecular Formula	*m*/*z* Theoretical	*m*/*z* Experimental	Error [ppm]	DBE	MS/MS Spectrum	Proposed Compound
1	[M-H]^−^	-	C_16_H_16_O_5_	287.0925	287.0928	−1.05	9	219.0294 190.0270 173.0241	Shikonin
2	[M]	-	C_21_H_24_O_7_	388.1522	388.1531	−2.31	10	270.0900 255.0650 179.0349 117.0542	Hydroxyisovalerylshikonin
3	[M]	-	C_18_H_18_O_6_	330.1103	330.1127	−7.15	10	270.0899 255.0655 237.0544 215.0347	Acetylshikonin
4	[M]	-	C_20_H_22_O_6_	358.1416	358.1432	−4.36	10	270.0909 255.0664 237.0562 215.0354	Isobutyrylshikonin
5	[M]	-	C_21_H_22_O_6_	370.1416	370.1426	−2.6	11	270.0905 255.0662 237.0574 215.0358	Dimethylacrylshikonin isomer 1
6	[M]	-	C_21_H_22_O_6_	370.1416	370.1423	−1.79	11	270.0900 255.0663 237.0562 215.0353	Dimethylacrylshikonin isomer 2
7	[M]	-	C_21_H_24_O_6_	372.1578	372.1587	−3.79	10	270.0896 255.0656 237.0553 215.0347	Isovalerylshikonin isomer 1
8	[M]	-	C_21_H_24_O_6_	372.1578	372.1565	−1.34	10	270.0907 255.0675 237.0555 215.0354	Isovalerylshikonin isomer 2

**Table 2:** Six-step exhaustive hexane extraction of shikonin derivatives from *Echium vulgare* (*n* = 3).

Six-Step Exhaustive Hexane Extraction	Yield mg/g DW ± SD	Shikonin Derivatives mg/g DW ± SD							
		1	2	3	4	5	6	7	8
Step 1	5.69 ± 0.91	0.017 ± 0.001	0.106 ± 0.011	0.642 ± 0.039	0.141 ± 0.014	0.043 ± 0.003	0.054 ± 0.006	0.220 ± 0.018	0.143 ± 0.011
Step 2	1.52 ± 0.22	0.007 ± 0.001	0.034 ± 0.006	0.169 ± 0.041	0.036 ± 0.009	0.012 ± 0.003	0.014 ± 0.002	0.055 ± 0.014	0.036 ± 0.009
Step 3	0.67 ± 0.04	0.004 ± 0.000	0.012 ± 0.002	0.048 ± 0.013	0.010 ± 0.003	n.d.	n.d.	0.014 ± 0.004	0.014 ± 0.004
Step 4	0.02 ± 0.01	0.002 ± 0.000	0.002 ± 0.000	0.011 ± 0.005	n.d.	n.d.	n.d.	n.d.	n.d.
Step 5	0.64 ± 0.11	0.003 ± 0.000	0.006 ± 0.000	0.018 ± 0.001	n.d.	n.d.	n.d.	n.d.	n.d.
Step 6	0.41 ± 0.06	0.002 ± 0.000	0.004 ± 0.000	0.011 ± 0.003	n.d.	n.d.	n.d.	n.d.	n.d.
Sum of extracts (1–6)	8.93	0.034	0.163	0.900	0.187	0.055	0.067	0.289	0.188
Sum of shikonins		1.884							

Shikonin derivatives: 1—shikonin; 2—hydroxyisovalerylshikonin; 3—acetylshikonin; 4—isobutyrylshikonin; 5—dimethylacrylshikonin isomer 1; 6—dimethylacrylshikonin isomer 2; 7—isovalerylshikonin isomer 1; 8—isovalerylshikonin isomer 2; n.d.—not detected.

**Table 3:** Six-step exhaustive TBa 2:8 extraction of shikonin derivatives from *Echium vulgare* (*n* = 3).

Six-Step Exhaustive TBa 2:8 Extraction	Yield mg/g DW ± SD	Shikonin Derivatives mg/g DW ± SD						
		2	3	4	5	6	7	8
Step 1	14.38 ± 1.13	0.175 ± 0.008	0.842 ± 0.029	0.171 ± 0.010	0.058 ± 0.003	0.062 ± 0.000	0.270 ± 0.010	0.172 ± 0.011
Step 2	6.68 ± 0.32	0.028 ± 0.001	0.111 ± 0.000	0.022 ± 0.001	n.d.	n.d.	0.035 ± 0.001	0.022 ± 0.002
Step 3	4.58 ± 0.38	0.009 ± 0.001	0.024 ± 0.001	n.d.	n.d.	n.d.	n.d.	n.d.
Step 4	4.37 ± 0.59	0.007 ± 0.00	0.011 ± 0.002	n.d.	n.d.	n.d.	n.d.	n.d.
Step 5	1.72 ± 0.053	0.006 ± 0.001	0.007 ± 0.001	n.d.	n.d.	n.d.	n.d.	n.d.
Step 6	1.75 ± 0.67	n.d.	n.d.	n.d.	n.d.	n.d.	n.d.	n.d.
Sum of extracts (1–6)	33.48	0.225	0.995	0.193	0.058	0.062	0.305	0.194
Sum of shikonins		2.032						

Shikonin derivatives: 1—shikonin; 2—hydroxyisovalerylshikonin; 3—acetylshikonin; 4—isobutyrylshikonin; 5—dimethylacrylshikonin isomer 1; 6—dimethylacrylshikonin isomer 2; 7—isovalerylshikonin isomer 1; 8—isovalerylshikonin isomer 2; n.d.—not detected.

**Table 4:** Amount (mg/g of dry extract) of shikonin derivatives in thymol:benzyl alcohol (molar ratio 2:8) and hexane dry extracts from *E. vulgare*.

Shikonin Derivatives	Solvent	
	TBa 2:8	Hexane
Shikonin	0.238	0.302
Hydroxyisovalerylshikonin	0.860	1.861
Acetylshikonin	3.823	11.29
Isobutyrylshikonin	0.783	2.481
Dimethylacrylshikonin isomer 1	0.311	0.765
Dimethylacrylshikonin isomer 2	0.359	0.944
Isovalerylshikonin isomer 1	1.163	3.864
Isovalerylshikonin isomer 2	0.775	2.506
Sum of shikonin	8.312	24.013

**Table 5:** Antimicrobial and antifungal activity of extracts *Echium vulgare* root extracts. Minimum inhibitory concentration (MIC), minimum bactericidal concentration (MBC), and minimum fungicidal concentration (MFC) values are expressed in mg dry extract/mL. Color intensity corresponds to MIC values; darker colors indicate lower MICs and higher antimicrobial activity, while lighter colors represent higher MICs.

Microorganisms	Hexane Extract		TBa 2:8 Extract	
Gram-positive bacteria	MIC	MBC	MIC	MBC
*Staphylococcus aureus* ATCC 29213	0.004	0.008	0.125	0.125
*Staphylococcus aureus* ATCC 25923	0.004	0.008	0.06	0.06
*Staphylococcus aureus* ATCC 6538	0.004	0.008	0.03	0.125
*Staphylococcus aureus* ATCC 43300	0.004	0.008	0.03	0.125
*Staphylococcus aureus* ATCC BAA1707	0.004	0.008	0.125	0.25
*Staphylococcus epidermidis* ATCC 12228	0.004	0.008	0.03	0.06
*Enterococcus faecalis* ATCC 29212	0.06	0.125	0.5	1
*Enterococcus faecalis* ATCC 51299	0.06	0.125	0.125	0.5
*Enterococcus faecium* ATCC 19434	0.03	0.06	0.25	0.5
*Micrococcus luteus* ATCC 10240	0.002	0.004	0.03	0.03
*Bacillus subtilis* ATCC 6633	0.002	0.004	0.06	0.125
*Bacillus cereus* ATCC 10876	0.008	0.03	0.125	0.25
*Bacillus cereus* ATCC 13061	0.03	0.125	0.125	0.5
Gram-negative bacteria	MIC	MBC	MIC	MBC
*Salmonella enteritidis* ATCC 13076	2	4	4	8
*Salmonella* Typhimurium ATCC 14028	0.5	4	2	8
*Proteus mirabilis* ATCC 12453	2	4	4	8
*Bordetella bronchiseptica* ATCC 4617	2	2	4	4
*Escherichia coli* ATCC 25922	2	4	4	8
*Escherichia coli* ATCC 35218	2	4	4	8
*Klebsiella pneumoniae* ATCC 13883	2	4	4	8
*Klebsiella pneumoniae* ATCC BAA 2146	2	4	4	8
*Enterobacter aerogenes* ATCC 13048	2	4	4	8
*Pseudomonas aeruginosa* ATCC 27853	2	2	4	4
*Pseudomonas aeruginosa* NIL	2	2	4	4
*Acinetobacter baumanii* ATCC 19606	2	2	4	4
Yeasts	MIC	MFC	MIC	MFC
*Candida albicans* ATCC 2091	0.25	0.5	1	4
*Candida albicans* ATCC 10231	0.25	0.5	1	4
*Candida auris* CDC B11903	0.25	1	1	2
*Candida glabrata* ATCC 90030	0.5	1	1	4
*Candida glabrata* ATCC 15126	0.5	0.5	1	2
*Candida parapsilosis* ATCC 22019	0.25	0.5	2	2
*Candida krusei* ATCC 14243	0.25	0.5	1	4
*Candida lusitaniae* ATCC 3449	0.25	0.5	2	4
*Candida tropicalis* ATCC 1369	0.25	1	1	4
*Geotrichum candidum* ATCC 34614	0.125	0.25	1	2
*Candida albicans* ATCC 14053	0.5	0.5	2	4
*Candida glabrata* ATCC 66032	0.5	1	2	2

## Data Availability

The dataset is available upon request from the authors.

## Supplementary Materials

The following supporting information can be downloaded at https://www.mdpi.com/article/10.3390/molecules31091434/s1, Figure S1. The MS/MS spectra of the tentatively identified metabolites present in the Table 1; Table S1. Comparison of compound losses (%) in hexane and TBa 2:8 extracts after lyophilization under different conditions; Table S2. Six-step exhaustive T:Ba in molar ratio 1:9 extraction of shikonin derivatives from *Echium vulgare* (*n* = 3); Table S3. Six-step exhaustive T:Ba in molar ratio 3:7 extraction of shikonin derivatives from *Echium vulgare* (*n* = 3); Table S4. Six-step exhaustive T:Ba in molar ratio 4:6 extraction of shikonin derivatives from *Echium vulgare* (*n* = 3); Table S5. Six-step exhaustive T:Ba in molar ratio 5:5 extraction of shikonin derivatives from *Echium vulgare* (*n* = 3); Table S6. Six-step exhaustive T:Ba in molar ratio 6:4 extraction of shikonin derivatives from *Echium vulgare* (*n* = 3).
