# Supplementary material for: Volatile Natural Deep Eutectic Solvents (VNADESs) for Extraction of Shikonin Derivatives from Echium vulgare Roots and Evaluation of Biological Activity
Source: Molecules. 2026 Apr 26;31(9):1434. doi: 10.3390/molecules31091434 (PMC13164743; doi:10.3390/molecules31091434)
Supplement: Supplementary file 1 [file molecules-31-01434-s001.zip › molecules-4252183-supplementary.pdf]

Shikonin

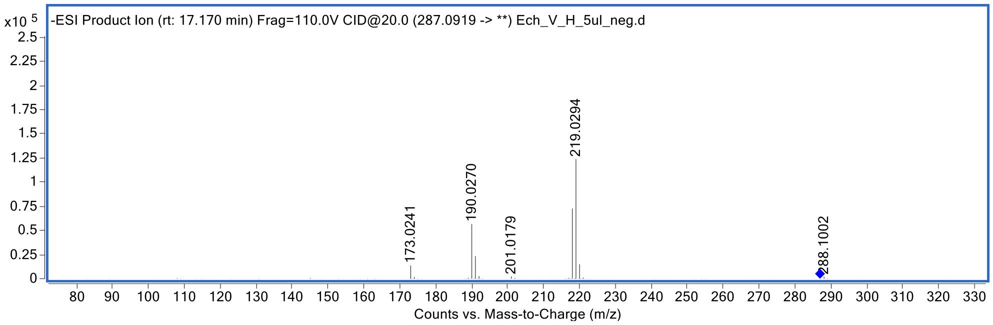

Hydroxyisovalerylshikonin

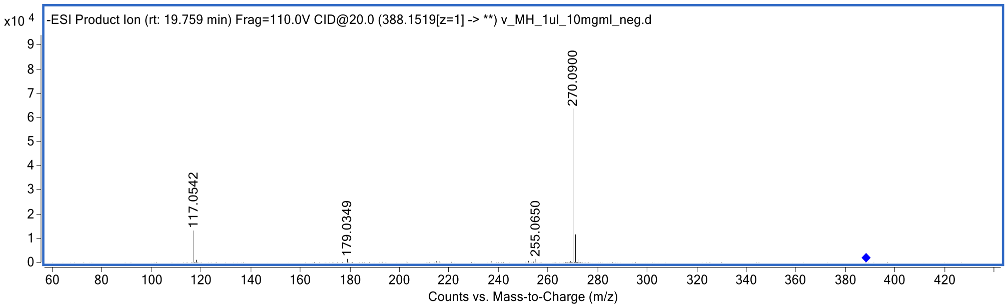

Acetylshikonin

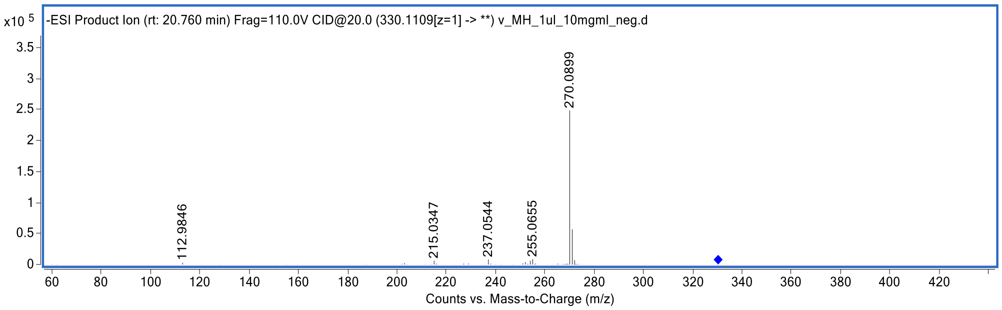

Isobutyrylshikonin

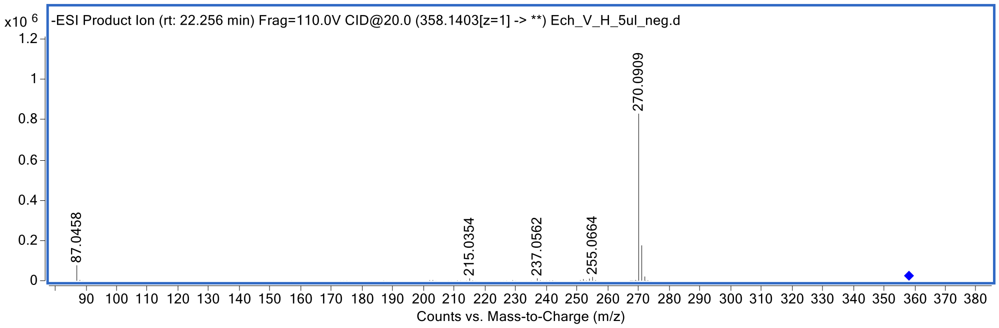

Dimetylacrylshikonin isomer 1

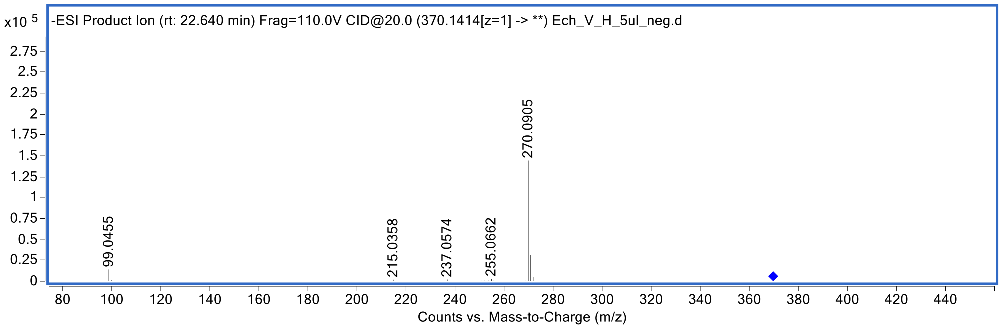

Dimetylacrylshikonin isomer 2

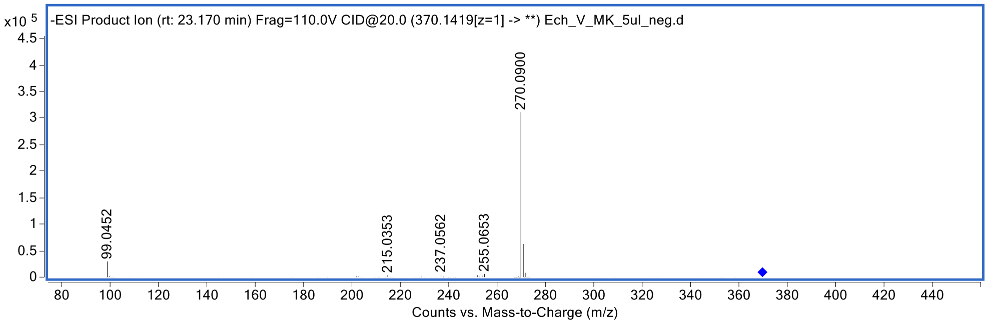

Isovalerylshikonin isomer 1

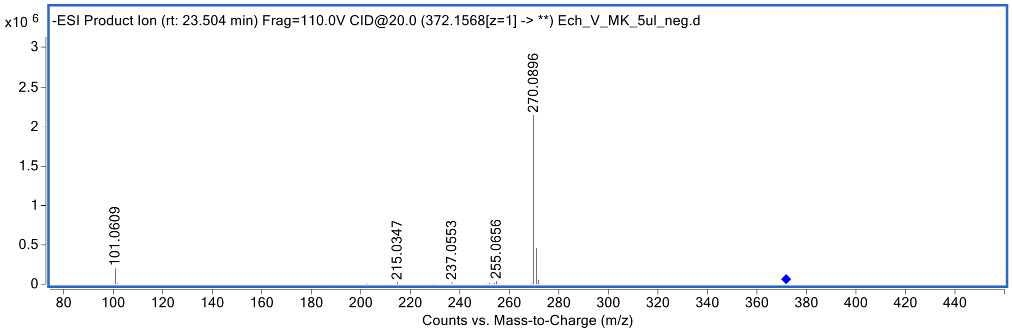

Isovalerylshikonin isomer 2

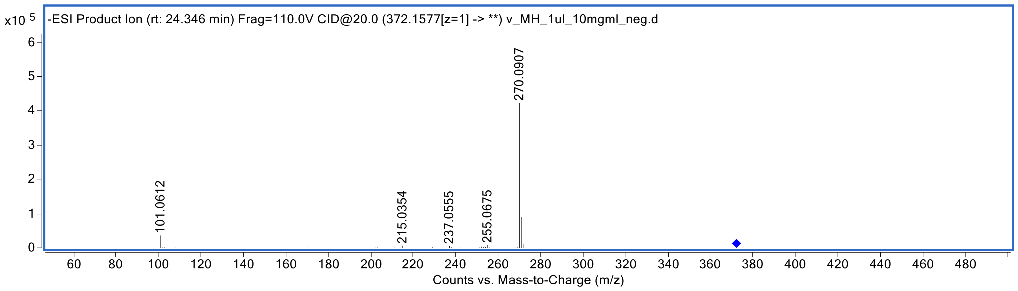

Figure S1. The MS/MS spectra of the tentatively identified metabolites present in the Table 1.

Table S1. Comparison of compound losses (%) in hexane and TBa 2:8 extracts after lyophilization under different conditions.

Shikonin derivative	TBa 2:8 extract (lyophilized to dryness/72h)	Hexane extract – 24 h	Hexane extract – 48 h	Hexane extract – 72 h
Shikonin (1)	-	64,28±3,74	81,62±4,45	80,86±3,17
Hydroxyisovalerylshikonin (2)	29,27±5,24	41,22±5,64	47,41±10,12	52,44±8,86
Acetylshikonin (3)	33,06±5,38	80,44±4,43	84,76±5,08	88,58±4,51
Isobutyrylshikonin (4)	34,14±6,37	62,70±7,87	63,48±9,60	73,62±10,62
Dimethylacrylshikonin isomer 1 (5)	23,12±5,48	35,47±4,32	50,60±12,04	58,89±3,04
Dimethylacrylshikonin isomer 2 (6)	16,16±3,58	50,20±10,71	54,66±4,36	60,88±12,52
Isovalerylshikonin isomer 1 (7)	38,08±5,54	39,29±3,81	51,21±8,68	59,50±15,75
Isovalerylshikonin isomer 2 (8)	35,27±6,54	29,63±8,64	49,93±14,50	56,28±11,05

Table S2. Six-step exhaustive T:Ba in molar ratio 1:9 extraction of shikonin derivatives from *Echium vulgare* (n=3)

Six-Step exhaustive T:Ba 1:9 extraction	Yield of the first extraction step (mg/g DW ± SD)	Shikonin derivatives (mg/g DW ± SD)						
		2	3	4	5	6	7	8
Step 1	15.09±1.26	0.170±0.008	0.816±0.048	0.166±0.010	0.056±0.006	0.056±0.007	0.265±0.018	0.166±0.005
Step 2		0.028±0.002	0.116±0.007	0.027±0.001	n.d.	n.d.	0.038±0.002	0.028±0.001
Step 3		0.009±0.000	0.028±0.0027	n.d.	n.d.	n.d.	n.d.	n.d.
Step 4		0.005±0.000	0.011±0.002	n.d.	n.d.	n.d.	n.d.	n.d.
Step 5		0.004±0.000	0.008±0.001	n.d.	n.d.	n.d.	n.d.	n.d.
Step 6		n.d.	n.d.	n.d.	n.d.	n.d.	n.d.	n.d.
Sum of extracts (1–6)		0.216	0.979	0.193	0.056	0.056	0.303	0.194
Sum of shikonins					1.996			

Shikonin derivatives: 2 – hydroxyisovalerylshikonin; 3 – acetylshikonin; 4 – isobutyrylshikonin; 5 – dimethylacrylshikonin isomer 1; 6 – dimethylacrylshikonin isomer 2; 7 – isovale-rylshikonin isomer 1; 8 – isovalerylshikonin isomer 2; n.d. – not detected.

Table S3. Six-step exhaustive T:Ba in molar ratio 3:7 extraction of shikonin derivatives from *Echium vulgare* (n=3)

Six-Step exhaustive T:Ba 3:7 extraction	Yield of the first extraction step (mg/g DW ± SD)	Shikonin derivatives (mg/g DW ± SD)						
		2	3	4	5	6	7	8
Step 1	14.53±1.40	0.169±0.006	0.802±0.039	0.166±0.009	0.056±0.005	0.065±0.007	0.266±0.016	0.174±0.004
Step 2		0.031±0.002	0.134±0.013	0.030±0.004	n.d.	n.d.	0.044±0.006	0.026±0.004
Step 3		0.008±0.001	0.024±0.001	n.d.	n.d.	n.d.	n.d.	n.d.
Step 4		0.007±0.001	0.014±0.006	n.d.	n.d.	n.d.	n.d.	n.d.
Step 5		n.d.	n.d	n.d.	n.d.	n.d.	n.d.	n.d.
Step 6		n.d.	n.d.	n.d.	n.d.	n.d.	n.d.	n.d.
Sum of extracts (1–6)		0.215	0.974	0.196	0.056	0.065	0.31	0.2
Sum of shikonins					2.016			

Shikonin derivatives: 2 – hydroxyisovalerylshikonin; 3 – acetylshikonin; 4 – isobutyrylshikonin; 5 – dimethylacrylshikonin isomer 1; 6 – dimethylacrylshikonin isomer 2; 7 – isovale-rylshikonin isomer 1; 8 – isovalerylshikonin isomer 2; n.d. – not detected.

Table S4. Six-step exhaustive T:Ba in molar ratio 4:6 extraction of shikonin derivatives from *Echium vulgare* (n=3)

Six-Step exhaustive T:Ba 4:6 extraction	Yield of the first extraction step (mg/g DW ± SD)	Shikonin derivatives (mg/g DW ± SD)						
		2	3	4	5	**6**	**7**	**8**
Step 1	15.06±0.11	0.167±0.008	0.794±0.043	0.165±0.011	0.054±0.007	0.061±0.007	0.261±0.013	0.167±0.001
Step 2		0.030±0.001	0.125±0.002	0.028±0.001	n.d.	n.d.	0.039±0.000	0.027±0.001
Step 3		0.009±0.001	0.027±0.003	n.d.	n.d.	n.d.	n.d.	n.d.
Step 4		0.006±0.000	0.011±0.001	n.d.	n.d.	n.d.	n.d.	n.d.
Step 5		n.d.	0.007±0.002	n.d.	n.d.	n.d.	n.d.	n.d.
Step 6		n.d.	n.d.	n.d.	n.d.	n.d.	n.d.	n.d.
Sum of extracts (1–6)		0.212	0.964	0.193	0.054	0.061	0.300	0.194
Sum of shikonins		1.978						

Shikonin derivatives: 2 – hydroxyisovalerylshikonin; 3 – acetylshikonin; 4 – isobutyrylshikonin; 5 – dimethylacrylshikonin isomer 1; 6 – dimethylacrylshikonin isomer 2; 7 – isovale-rylshikonin isomer 1; 8 – isovalerylshikonin isomer 2; n.d. – not detected.

Table S5. Six-step exhaustive T:Ba in molar ratio 5:5 extraction of shikonin derivatives from *Echium vulgare* (n=3)

Six-Step exhaustive T:Ba 5:5 extraction	Yield of the first extraction step (mg/g DW ± SD)	Shikonin derivatives (mg/g DW ± SD)						
		2	3	4	5	6	7	8
Step 1	14.46±1.13	0.167±0.006	0.810±0.035	0.164±0.009	0.060±0.004	0.069±0.011	0.261±0.016	0.168±0.004
Step 2		0.026±0.004	0.112±0.021	0.022±0.003	n.d.	n.d.	0.033±0.006	0.020±0.002
Step 3		0.009±0.000	0.029±0.002	n.d.	n.d.	n.d.	n.d.	n.d.
Step 4		0.007±0.000	0.009±0.000	n.d.	n.d.	n.d.	n.d.	n.d.
Step 5		0.006±0.000	0.010±0.001	n.d.	n.d.	n.d.	n.d.	n.d.
Step 6		n.d.	n.d.	n.d.	n.d.	n.d.	n.d.	n.d.
Sum of extracts (1–6)		0.215	0.97	0.186	0.060	0.075	0.294	0.188
Sum of shikonins		1.982						

Shikonin derivatives: 2 – hydroxyisovalerylshikonin; 3 – acetylshikonin; 4 – isobutyrylshikonin; 5 – dimethylacrylshikonin isomer 1; 6 – dimethylacrylshikonin isomer 2; 7 – isovale-rylshikonin isomer 1; 8 – isovalerylshikonin isomer 2; n.d. – not detected.

Table S6. Six-step exhaustive T:Ba in molar ratio 6:4 extraction of shikonin derivatives from *Echium vulgare* (n=3)

Six-Step exhaustive T:Ba 6:4 extraction	Yield of the first extraction step (mg/g DW ± SD)	Shikonin derivatives (mg/g DW ± SD)						
		2	3	4	5	6	7	8
Step 1	14.32±1.52	0.156±0.009	0.747±0.048	0.159±0.010	0.052±0.002	0.058±0.009	0.242±0.018	0.155±0.011
Step 2		0.028±0.001	0.115±0.000	0.025±0.000	n.d.	n.d.	0.036±0.002	0.022±0.001
Step 3		0.010±0.000	0.027±0.002	n.d.	n.d.	n.d.	n.d.	n.d.
Step 4		0.007±0.002	0.012±0.001	n.d.	n.d.	n.d.	n.d.	n.d.
Step 5		0.006±0.000	0.008±0.000	n.d.	n.d.	n.d.	n.d.	n.d.
Step 6		n.d.	n.d.	n.d.	n.d.	n.d.	n.d.	n.d.
Sum of extracts (1–6)		0.207	0.909	0.184	0.052	0.058	0.278	0.177
Sum of shikonins					1.865			

Shikonin derivatives: 2 – hydroxyisovalerylshikonin; 3 – acetylshikonin; 4 – isobutyrylshikonin; 5 – dimethylacrylshikonin isomer 1; 6 – dimethylacrylshikonin isomer 2; 7 – isovalerylshikonin isomer 1; 8 – isovalerylshikonin isomer 2; n.d. – not detected.
